# Phage resistance of *Bordetella avium* due to an altered cell wall results in increased susceptibility to the polypeptide antibiotics colistin and polymyxin B

**DOI:** 10.1038/s41598-025-30405-7

**Published:** 2025-12-19

**Authors:** Dorothee Serian, Yury Churin, Jens André Hammerl, Arne Jung, Min Yue, Anja Müller, Corinna Kehrenberg

**Affiliations:** 1 Institute for Veterinary Food Science, Faculty of Veterinary Medicine, Justus Liebig University Giessen, Frankfurter Str. 92, 35392 Giessen, Germany; 2 Department Biological Safety, German Federal Institute for Risk Assessment, Berlin, Germany; 3 Clinic for Poultry, University of Veterinary Medicine Hannover Foundation, Hannover, Germany; 4 Institute of Preventive Veterinary Science and Department of Veterinary Medicine, Zhejiang University College of Animal Sciences, Hangzhou, China; 5 Hainan Institute of Zhejiang University, Sanya, China

**Keywords:** *Bordetella avium*, Phage resistance, Bacterial membrane, LPS, Antimicrobial susceptibility, Bacteriology, Bacteriophages, Microbiology, Antimicrobial resistance

## Abstract

Infections caused by multidrug-resistant bacterial pathogens are becoming increasingly frequent, so alternative therapeutic strategies such as phage therapy are gaining in importance. However, one of the main challenges in phage therapy is the rapid emergence of phage-resistant bacteria. Hence, this study investigated the occurrence of phage resistance in *Bordetella avium*, the causative agent of bordetellosis in poultry of all ages, with a special focus on the association between bacterial phage resistance and antibiotic resistance. Here, we describe the isolation and analysis of four *B. avium* mutants that showed resistance to *B. avium* phages, with decreased adsorption leading to phage resistance of the mutants. SDS-PAGE analysis revealed a lipopolysaccharide (LPS) structure with altered nuclear patterns and a missing O-antigen moiety in the phage-resistant *B. avium* mutants. Whole-genome sequence analysis revealed mutations in the genetic loci BAV2233 and BAV2235, encoding lipopolysaccharide glucosyltransferase family 4 proteins, and BAV0511, encoding Vi polysaccharide biosynthesis UDP-N-acetylglucosamine C-6 dehydrogenase TviB protein, which are involved in LPS biosynthesis. Additionally, antimicrobial susceptibility testing against nine antibiotics using the agar dilution method showed that LPS changes in phage-resistant mutants affected the susceptibility of *B. avium* to two of the antibiotics examined. Specifically, the MIC values of the polymyxin antibiotics colistin and polymyxin B decreased by up to five two-fold dilutions, depending on the isolate considered. An association was observed between the length of the polysaccharide chain in LPS and the susceptibility of *B. avium* to colistin and polymyxin B, with shorter chains indicating higher susceptibility. Collective findings illuminate a beneficial aspect that may support the use of *B. avium* phages. Based on these results, genetically modified phages based on these *B. avium* phages would be a promising direction for future phage applications as an alternative to the use of antimicrobial agents.

**Supplementary Information:**

The online version contains supplementary material available at 10.1038/s41598-025-30405-7.

## Introduction

The development of antimicrobial agents for therapeutic purposes is a remarkable achievement of modern medicine. Antibiotics play a crucial role in reducing the morbidity and mortality caused by numerous bacterial infections in humans and in animals. However, bacteria have evolved diverse mechanisms to counteract the effects of antibiotics, enabling them to develop or acquire antibiotic resistance^1^. The increasing mortality rate associated with infections caused by antibiotic-resistant bacteria worldwide, as reported annually by the World Health Organization (WHO), is a cause of great concern^2^. Therefore, alternative treatment approaches for bacterial infections, such as bacteriophage therapy, have gained increasing attention. Bacteriophages (phages) are viruses that have the ability to infect bacteria and specifically lyse them. The use of phages as therapeutic agents has already been studied, highlighting both their advantages and disadvantages^3^. One disadvantage of phages for therapy, similar to antimicrobial agents, is that they can encounter bacterial pathogens that have developed resistance to phages^4,5^.

*Bordetella avium*, a Gram-negative bacterium, is the causative agent of bordetellosis, a frequently occurring respiratory disease in poultry that leads to significant economic losses in commercial poultry farms worldwide^6,7^. The current treatment methods are primarily based on the use of antimicrobial agents such as macrolides, tetracyclines, trimethoprim-sulfonamide combinations and fluoroquinolones. However, resistance to these agents is being reported more and more frequently^7–9^. Consequently, the potential use of phages as a future treatment approach for bordetellosis is attracting interest.

In Gram-negative bacteria, lipopolysaccharides (LPS) serve as part of the double-membrane barrier called the outer membrane (OM)^10^. LPS is an OM component found in most Gram-negative bacteria, although not in all of them^11^. The LPS has various biological functions in bacteria and is exclusively located in the outer leaflet of the OM in *B. avium* and other Gram-negative bacteria. LPS molecules are primarily composed of a conserved lipid A moiety to which core and outer polysaccharides are typically bound. In most cases, lipid A is glycosylated with a core oligosaccharide that can serve as a binding site for long-chain O-antigenic polysaccharides (O-antigens)^12,13^. Bacterial LPS, which includes an O-antigen component, is referred to as smooth, whereas LPS lacking the O-antigen is denoted as rough^14^.

Resistance mechanisms against phages are attributed to various bacterial cell wall modifications and alterations in intracellular metabolic pathways. Examples are superinfection exclusion systems that prevent phage DNA entry or editing of foreign DNA using clustered regularly interspaced short palindromic repeats (CRISPR), as well as nucleic acid restriction-modification systems (R-M) that can cleave phage DNA^5,15,16^. LPS acts as a specific receptor for many phages on the bacterial surface, and modifications of LPS have the potential to lead to phage resistance by affecting specific interactions between phages and bacteria^15,17,18^.

In a previous study, we isolated and characterized seven temperate LPS-binding *B. avium* phages from the poultry environment with a broad host range^19^. The phages exhibit a lytic phenotype, although they contain genes that encode putative markers for lysogeny. Our research showed that the phages can indeed undergo a lysogenic life cycle with varying frequency, but the lysogenic bacteria remained susceptible to superinfection with the same phages^19^. Here, we describe the isolation and analysis of phage-resistant *B. avium* mutants. These mutants possess mutations in the genetic loci involved in LPS biosynthesis, which resulted in the accumulation of truncated LPS. Bacteria with modified LPS are more susceptible to the polypeptide antibiotics colistin and polymyxin B, with susceptibility depending on the length of the polysaccharide chains in the LPS structure.

## Results

### Isolation and characterization of phage-resistant mutants

A recent study showed that *B. avium* phages vB_BaM-IFTN1-7 (IFTN1-7) produced clear lysis spots on 50 different *B. avium* field isolates and the *B. avium* type strain CCUG 13726^ T^ after 20 h of incubation^19^. However, after incubation for 48 h, growth of single colonies on the previously clear lysed spots was observed. Therefore, in the course of the present study, single colonies were collected from the spot zones of the following isolate-phage combinations: *B. avium* isolate 12/574/1/C infected with phage IFTN3, and *B. avium* type strain CCUG 13726^ T^, isolate × 1760, and isolate × 590/1a infected with phage IFTN4. The two phages were selected because they differed in their host range, as previously shown^19^. After two passages of culturing these colonies on TSA agar plates, phage reinfection was tested. The spot assay demonstrated no lysis receptivity of the isolated bacterial colonies when using the seven *B. avium* phages, IFTN1 to IFTN7, indicating phage resistance (Fig. S1).

Growth curves of phage-resistant *B. avium* were compiled and compared with the growth curves of the phage-susceptible parent isolates. However, all phage-resistant mutants showed similar growth characteristics in the lag, log, stationary, and death phases compared to the parent *B. avium* isolate (Fig. S2). This finding prompted further investigations to determine whether the growth of phage-resistant bacteria within the same broth culture could affect the growth of phage-susceptible *B. avium.* Therefore, growth competition experiments were performed. As presented in Figure S3, all phage-resistant mutants tended to have higher growth rates (at least after 72 h) than the parental isolates. However, the difference was not statistically significant.

### SDS-PAGE analysis of LPS from phage-resistant mutants

**Fig. 1:**
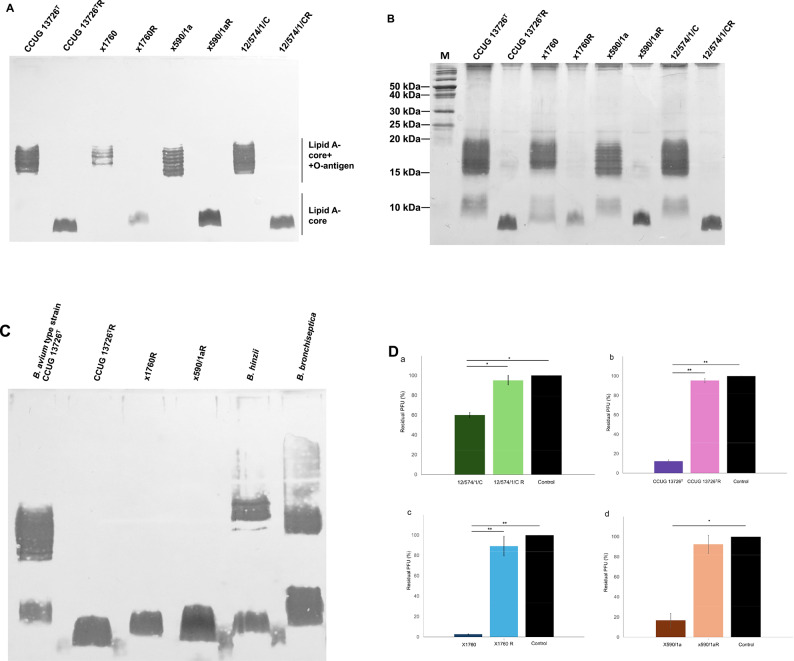
SDS-PAGE profile of *B. avium* LPS and adsorption assay. (**A**) LPS extracted from *B. avium* type strain CCUG 13726^ T^ and three field isolates, × 1760, × 590/1a, 12/574/1/C, and their corresponding phage-resistant mutants, were separated by SDS-PAGE and visualised by silver staining. (**B**) The gel with the same samples as in (A) was developed longer to demonstrate the short LPS molecular species in the original isolates. (**C**) Comparison of LPS profiles of *Bordetella* strains(**D**) The adsorption of phage vB_BaM-IFTN3 to *B. avium* isolate 12/574/1/C and to the phage-resistant mutant 12/574/1/CR (a), and of phage vB_BaM-IFTN4 to *B. avium* type strain CCUG 13726^ T^ (b), *B. avium* isolate × 1760 (c), and *B. avium* isolate × 590/1a (d), and to the phage-resistant mutants CCUG 13726^T^R (b), x1760R (c), and × 590/1aR (d) are shown (mean ± SD). * *P* < 0.05, ** *P* < 0.025. Control samples without pre-incubation with bacteria were included. The results represent the average of three independent experiments.

To determine whether LPS was altered in phage-resistant mutants, we performed SDS-PAGE analysis and silver staining of LPS from the phage-resistant mutants and their parental strains (Fig. 1A, B). For better visibility, the gel in Fig. 1B was subjected to silver staining for a longer period of time than the gel shown in Fig. 1A, resulting in more pronounced bands. For uniform presentation and to focus on the relevant parts, parts of the images have been slightly cropped and the images are in black and white, but the original images can be found in the Supplementary Information file (Appendix, original images). All mutants demonstrated a prominent band with high mobility in the SDS-PAGE gel, indicating a truncation of LPS, resulting in a rough LPS form^12,14^.

To estimate the approximate size of LPS molecules from *B. avium* mutants, they were compared with LPS prepared from *Bordetella hinzii* and *Bordetella bronchiseptica* isolates (Fig. 1C). The short-chain molecules of LPS from *B. hinzii* are composed of a lipid A moiety and a free core oligosaccharide^20^. *B. bronchiseptica* express short-chain molecules of LPS composed of a lipid A moiety plus a free core oligosaccharide but also molecules that are further modified by the addition of a complex trisaccharide^21^. LPS from *B. avium* mutants ran in the gel at the same level as O-antigen chain-free LPS from *B. hinzii* (Fig. 1C). LPS from CCUG 13726^T^R moved slightly slower than short LPS molecules from *B. hinzii*, while LPS from mutants x1760R and × 590/1aR migrated at the same level as short LPS from *B. hinzii* (Fig. 1C) in the SDS-PAGE gel^20^.

### Decreased adsorption resulted in phage resistance of *B. avium* mutants

The adsorption of phages onto host receptors marks the beginning of phage infection. Phage resistance can occur through various mechanisms, including mutations that alter the cell surface, such as truncation of LPS structures^5,22^. Therefore, adsorption assays were conducted to evaluate whether the adsorption of phages IFTN3 and IFTN4 was impeded in the *B. avium* mutants (Fig. 1D). The assays showed that a significantly lower percentage of residual plaques occurred after incubation of the phages with the parental *B. avium* isolates compared to the phage-resistant mutants. This suggested that phage-resistant mutants did not adsorb phages, or adsorb only minimal amounts. This alteration was observed for all four phage-resistant mutants (Fig. 1D). Taken together, these findings indicated that the development of phage resistance was due to changes in the LPS structures of the phage-resistant mutants, which inhibited phage adsorption.

### Comparative genomics of *B. avium* strains and their phage-resistant mutants

To identify the genetic mutations leading to phage resistance, we conducted comparative analyses of the genomes of *B. avium* parental strains and their phage-resistant mutants. Whole-genome sequencing of all four phage-resistant mutants, as well as of isolates × 1760 and × 590/1a, was performed. The genome sequences of *B. avium* type strain CCUG 13726^ T^ (accession number QVSW00000000, and NZ_UFTG00000000) and isolate 12/574/1/C (QVSX00000000) were obtained from the National Center for Biotechnology Information (NCBI)^23,24^. Snippy v4.6.0^25^ was used to identify single nucleotide polymorphisms (SNPs) between genomes and genome comparisons were done using progressiveMauve^26^. The coverage of variant calls from different phage-resistant mutants determined with Snippy was for *B. avium* CCUG 13726TRC3: A:70 – ACATCAT:0, for *B. avium* X590/1aR AC:68 – A:0, for *B. avium* 12/574/c/1 T:67 – C:0, and for *B. avium* 12/574/C/1: T:89 – C:0. The mutations in *B. avium* CCUG 13726TR and CCUG 13726TRC1 were discovered by genome comparison with progressiveMauve^26^. All genetic variations found in the phage-resistant mutants were located in genes involved in the biosynthesis of LPS (Table 1). No large chromosomal deletions or rearrangements were found in the phage-resistant mutants.

**Table 1:** Genetic variations identified in phage-resistant *B. avium* mutants.

Mutant	Nucleotide position*	Mutation or nucleotide sequence alteration^a^	Protein function
CCUG 13726^T^R	2,406,718	Insertion of CTGC; resulting in frameshift	BAV2233; glycosyl transferase family 4 protein
CCUG 13726^T^RC3	98,411	Stop-gained disruptive in-frame deletion of CATCAT	BAV0096; pyridoxal phosphate-dependent aspartate aminotransferase
CCUG 13726^T^RC1	85,358	Substitutions: nucleotide C → T amino acid S159L	BAV0086; glycosyl transferase family 2 protein
x1760R	527,627	Insertion of C; resulting in frameshift	BAV0511; Vi polysaccharide biosynthesis UDP-N-acetylglucosamine C-6 dehydrogenase TviB
× 590/1aR	2,409,160	Substitutions: nucleotide C → T amino acid H9Y	BAV2235; glycosyltransferase family 4 protein
12/574/1/CR	2,406,134	Substitutions: nucleotide C → T amino acid Q373*	BAV2233; glycosyltransferase family 4 protein

*Nucleotide position in the genome of *B. avium* strain 197N.

^a^Genome sequences from the parent strains were used for genome comparisons with mutants.

Because all *B. avium* studies on LPS were carried out with *B. avium* 197N^20,27–29^, and the sequence of this strain has been fully annotated and published^22^, we decided to use the designations for the genomic loci for this strain. Two of the detected nucleotide sequence alterations were located at the same gene locus (BAV2233 in *B. avium* 197 N)^22^, which encodes a glycosyltransferase-family-4 protein. One was a four-nucleotide insertion (CTGC) that resulted in a frame shift in *B. avium* CCUG 13726^T^R, and the other was a nonsense mutation C → T that resulted in a premature stop codon in 12/574/1/CR. Another nucleotide sequence alteration was the insertion of a C in the locus encoding the Vi polysaccharide biosynthesis UDP-N-acetylglucosamine C-6 dehydrogenase TviB (BAV0511 in *B. avium* 197 N), resulting in a frameshift in the mutant x1760R. In the fourth phage-resistant *B. avium* mutant, × 590/1aR, a missense mutation, C → T, was detected in the locus encoding a glycosyltransferase-family-4 protein (BAV2235 in *B. avium* 197 N), which led to an amino acid substitution of H9Y.

**Fig. 2:**
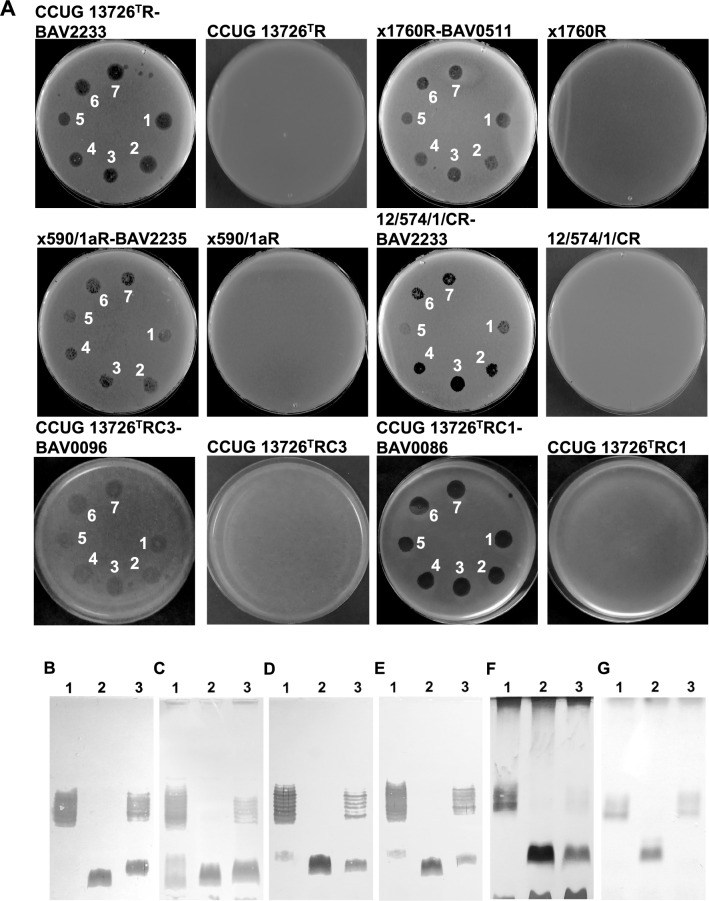
Complementation analysis of *B. avium* phage-resistant mutants. (A) Spot test of *B. avium* phages on phage-resistant and complemented mutants. 1–7- phages vB_BaM-IFTN1-7. LPS profiles of the complemented phage-resistant *B. avium* mutants. (**B**) 1- type strain CCUG 13726^ T^, 2- CCUG 13726^T^R, 3- CCUG 13726^T^R complemented with the plasmid pUR containing the locus BAV2233 (pUR-BAV2233); (**C**) 1- × 1760, 2- x1760R, 3- x1760R complemented with the plasmid pUR-BAV0511; (D) 1- × 590/1a, 2- × 590/1aR, 3- × 590/1aR complemented with the plasmid pUR-BAV2235; (**E**) 1- 12/574/1/C, 2- 12/574/1/CR, 3- 12/574/1/CR complemented with the plasmid pUR-BAV2233; (**F**) 1- type strain CCUG 13726^ T^, 2- CCUG 13726^T^RC3, 3- CCUG 13726^T^RC3 complemented with the plasmid pUR-BAV0096; (**G**) 1- type strain CCUG 13726^ T^, 2- CCUG 13726^T^RC1, 3- CCUG 13726^T^RC1 complemented with the plasmid pUR-BAV0086. The sub-sections B-G show the results from different gels.

To confirm that the observed genetic variations in the mutants were responsible for the phage-resistant phenotype, we performed complementation experiments. Plasmids containing wild-type alleles of each mutated gene were introduced into the mutant cells by conjugation. We found that the expression of the wild-type alleles restored phage susceptibility (Fig. 2A), indicating that these mutations were indeed responsible for the phage-resistant phenotype. Additionally, we observed that the mutants produced smooth LPS structures after complementation (Fig. 2B-G), even though the change in LPS patterns was only slightly detectable in one isolate (Fig. 2F). This suggested that the wild-type alleles introduced by the plasmids were able to restore the function of the mutated genes.

### Reduced swimming motility of *B. avium* phage-resistant mutants

It has been previously shown that *Salmonella enterica*^30^ and *Pseudomonas aeruginosa*^31^ require LPS biosynthesis to move on swimming motility plates. Phage-resistant mutants of *B. avium*, however, exhibited reduced swimming motility (Table S3 and Fig. S4). We observed significant differences in swimming zone diameters between *B. avium* type strain CCUG 13726^ T^ after 24 h of incubation at 37 °C and its phage-resistant mutant CCUG 13726^T^R, between *B. avium* × 1760 and its mutant x1760R, and between *B. avium* 12/574/1/C and its mutant 12/574/1/CR. However, the difference was not significant for isolate *B. avium* × 590/1a and its mutant × 590/1aR.

### Increased susceptibility of phage-resistant *B. avium* mutants to polypeptide antibiotics

To determine the antimicrobial susceptibility of *the B. avium* parental isolates and their phage-resistant mutants, all isolates were subjected to agar dilution susceptibility testing according to CLSI document VET01, including the antimicrobial agents ampicillin (AMP), ceftazidime (CAZ), ciprofloxacin (CIP), colistin (CST), kanamycin (KAN), sulfafurazole (SUL), trimethoprim (TMP), tetracycline (TET), and polymyxin B (PMB)^32^ (Tables 2 and S1). The phage-resistant mutants CCUG 13726^T^R and 12/574/1/CR revealed increased susceptibility, as indicated by a 16-fold decrease in their MIC values of CST and PMB (0.06 µg/ml), compared to their parent strains (1.0 µg/ml). For × 590/1aR and x1760R, the MIC values were 0.125 µg/ml for CST and 0.25 µg/ml for PMB, representing a 16- and eightfold MIC reduction for x1760R and an 8- and fourfold reduction in the case of mutant × 590/1aR, respectively (Table 2). Due to the lack of approved breakpoints for *B. avium*, it was not possible to classify the isolates as susceptible or resistant. By introducing the wild-type allele into the mutant cells, the MIC values for both the antibiotics colistin and polymyxin B were restored nearly to the level of the parent strains (Table 2). In summary, the phage-resistant mutants showed increased susceptibility to CST and PMB of up to five MIC dilution steps compared to the parent strains.

**Table 2:** MIC values of *B. avium* isolates and their phage-resistant mutants.

Isolate	MIC (µg/ml)^a^	
	CST	PMB
CCUG 13726^ T^	1.0	1.0
CCUG 13726^T^R^b^	0.06	0.06
CCUG 13726^T^R::BAV2233^c^	0.5	1.0
CCUG 13726^T^RC3^d^	0.5	0.5
CCUG 13726^T^RC1^d^	1.0	1.0
CCUG 13726^T^ΔBAV2230	0.03	0.03
12/574/1/C	1.0	1.0
12/574/1/CR^b^	0.06	0.06
12/574/1/CR::BAV2233	0.5	1.0
× 590/1a	1.0	1.0
X590/1aR^b^	0.125	0.25
X590/1aR::BAV2235	0.5	0.5
× 1760	2.0	2.0
x1760R^b^	0.125	0.25
x1760R::BAV0511	2.0	2.0

CST- colistin, PMB- polymyxin B.

^a^minimal inhibitory concentration.

^b^the suffix R indicates phage-resistant mutants.

^c^these mutants were complemented with the respective gene.

^d^RC mutants were selected at low frequency on colistin supplemented agar plates.

### The role of LPS in the susceptibility of *B. avium* to colistin and polymyxin B

**Fig. 3:**
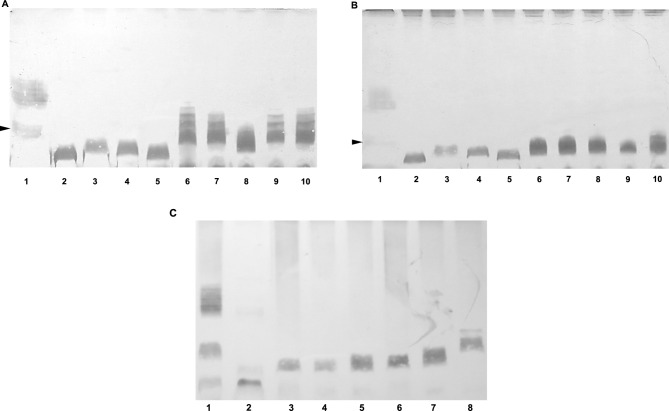
SDS-PAGE profile of LPS extracted from phage-resistant *B. avium* mutants that did not show reduced susceptibility to colistin or polymyxin B. (**A**) 1- *B. avium* type strain CCUG 13726^ T^, 2- CCUG 13726^T^R, 3- x1760R, 4- × 590/1aR, 5- 12/574/1/CR, 6- CCUG 13726^T^RC1, 7- CCUG 13726^T^RC2, 8- CCUG 13726^T^RC3, 9- CCUG 13726^T^RC4. Mutants shown in lanes 6 to 9 were selected on colistin-supplemented agar plates. (**B**) 1- *B. avium* type strain CCUG 13726^ T^, 2- CCUG 13726^T^R, 3- x1760R, 4- × 590/1aR, 5- 12/574/1/CR, 6- CCUG 13726^T^RP1, 7- CCUG 13726^T^RP2, 8- CCUG 13726^T^RP3, 9- CCUG 13726^T^RP4. Mutants shown in lanes 6 to 9 were selected on polymyxin B-supplemented agar plates. Arrows indicate Lipid A core oligosaccharides. (**C**) SDS-PAGE profile of LPS from phage-resistant *B. avium*. LPS extracted from *B. avium* type strain CCUG 13726^ T^ (1) and phage-resistant mutants CCUG 13726^T^ΔBAV2230 (2), CCUG 13726^T^R (3), 12/574/1/CR (4), X590/1aR (5), x1760R (6), CCUG 13726^T^RC3 (7), CCUG 13726^T^RC1 (8) were separated by SDS-PAGE and visualized by silver staining.

*B. avium* mutants demonstrated low MIC values of CST and PMB (Table 2). Therefore, we performed experiments to determine whether it is possible to isolate mutants that are resistant to phages, but do not exhibit increased susceptibility to CST and PMB. It turned out that such mutants occurred, but with a low frequency of 3.78 × 10^–4^ (± 0.65 × 10^–4^) for CST and 3.91 × 10^–4^ (± 2.17 × 10^–4^) for PMB. SDS-PAGE analysis of their LPS profiles (n = 16 mutants) revealed that these phage-resistant mutants (indicated with suffix RC or RP, respectively) possessed a rough-type LPS that migrated in the region of lipid A linked to the core of the *B. avium* type strain CCUG 13726^ T^ LPS (Fig. 3A, B). However, there were differences in the mobility of LPS from various mutants. LPS with a smaller molecular weight migrated faster than the *B. avium* type strain CCUG 13726^ T^ core-linked lipid A and slower than LPS from mutants x1760R and × 590/1aR (Fig. 3A, B). Thus, the polysaccharide chain length of LPS appeared to determine the susceptibility of *B. avium* to colistin and polymyxin B.

We performed sequence analysis of the mutants CCUG 13726^T^RC3 (line 8, Fig. 3A) and CCUG 13726^T^RC1 (line 6, Fig. 3A). Analysis with Snippy v4.6.0^25^ showed that the mutation in strain CCUG 13726^T^RC3 was a disruptive in-frame deletion of CATCAT in gene locus BAV0096^22^, which encodes a pyridoxal phosphate-dependent aspartate aminotransferase. In contrast, analysis of the nucleotide sequence of mutant CCUG 13726TRC1 revealed a C → T nucleotide substitution leading to amino acid substitution S159L in the BAV0086 locus^22^, which encodes a glycosyltransferase family 2 protein. Both mutants exhibited a rough LPS form that either showed a similar migration pattern to the fast migrating molecular species in strain CCUG 13726^ T^ (in the case of CCUG 13726^T^RC1) or moved slightly faster (CCUG 13726^T^RC3) (Fig. 3A). In addition, susceptibility testing showed that the CCUG 13726^T^RC1 and RC3 mutants exhibited markedly higher MIC values of CST and PMB than the R mutant (Table 2). Furthermore, our results showed that expression of wild-type alleles of the mutated genes in RC1 and RC3 *B. avium* restored phage susceptibility (Fig. 2A), and resulted in smooth LPS structures after complementation (Fig. 2B-G).

To obtain a strain with an even shorter LPS than in the mutants CCUG 13726^T^R and 12/574/1/CR (the strains with the shortest LPS obtained by selection of phage-resistant mutants), we inactivated the ORF corresponding to BAV2230, which (in the genome of *B. avium* 197 N) encodes an ADP-heptose LPS heptosyltransferase II. This transferase is assumed to be responsible for the synthesis of the inner core of LPS^22,28^. Indeed, we observed that LPS isolated from the mutant CCUG 13726^T^Δ2230 migrated faster than LPS from CCUG 13726^T^R and 12/574/1/CR (Fig. 3C). This mutant was more susceptible to CST and PMB (MIC values 0.03 µg/ml for both antibiotics) (Table 2).

### Testing membrane permeability via susceptibility testing with rifampicin and novobiocin

It has been previously shown that mutants of LPS biosynthesis become hypersusceptible to antibiotics, such as novobiocin and rifampicin, as a result of OM perturbation^33,34^. Therefore, *B. avium* parental isolates and their phage-resistant mutants were additionally subjected to susceptibility testing against novobiocin (NV) and rifampicin (RD) (Table S2). All selected mutants had the same or even higher MIC values than those of the parent isolates. Only the inactivation of ORF2230 resulted in significantly higher susceptibility (eightfold MIC reduction) to both antibiotics. Thus, truncation of the inner core region of LPS in *B. avium* resulted in an abrupt loss of the major permeability barrier function of LPS while simultaneously causing only a gradual increase in susceptibility to CST and PMB compared to the CCUG 13726^T^R mutant.

## Discussion

In the fear of a post-antibiotic era, interest in bacteriophages is currently increasing. However, bacteria can develop resistance not only to antibiotics but also to phages^5^. In this study, we isolated phage-resistant mutants of four *B. avium* isolates on solid agar plates. In a previous study, increases in OD values were also observed during co-cultivation in liquid medium, indicating the development of resistant mutants after several hours of incubation^19^. Phage resistance occurred because of mutations in the genes responsible for LPS biosynthesis, resulting in LPS modification. Interestingly, these mutants exhibited increased susceptibility to colistin and polymyxin B.

To determine genetic variations of phage-resistant *B. avium* isolates, the gene loci were aligned with those from *B. avium* 197 N (GenBank accession no. AM167904). Because the genes involved in LPS biosynthesis have been investigated in several studies on *Bordetella* species *pertussis* and *bronchiseptica*, these two *Bordetella* species were also included in the analyses^20,29,35^. It was shown that the mutation in the phage-resistant *B. avium* × 590/1aR was located in a gene locus, that is (in the parental strain × 590/1a) 100% identical to BAV2235 from *B. avium* 197 N (Table 1). There is also similarity to BP2328 in *B. pertussis* (CAE42601), with the deduced proteins having 72% amino acid identity and 80% similarity^35^. Caroff et al. 2000 described the structure of the lipooligosaccharide (LOS) of *B. pertussis* as consisting of lipid A linked to a highly branched dodecasaccharide^36^. The gene locus BP2328 belongs to the LOS core biosynthesis gene cluster and encodes a protein that functions as a glucosamine, glucose-α1,4-glucosaminyltransferase^37^. Guertsen et al. 2009 described a *B. pertussis* BP2328 mutant strain with a complete core LOS structure that lacks five sugar residues, and thus consists of only seven sugar residues^37^. It can be concluded from these studies that the proteins encoded by the open reading frames (ORFs), BAV2235 from *B. avium* 197 N and BP2328 from *B. pertussis*, likely play similar roles in LPS biosynthesis. Based on the mutation in the phage-resistant *B. avium* × 590/1aR isolated in this study in the gene region corresponding to BAV2235 from *B. avium* 197 N and BP2328 in *B. pertussis*, it can be assumed that the LPS in × 590/1aR most likely contains lipid A and only seven sugar residues.

The mutation in *B. avium* x1760R was located at a locus corresponding to BAV0511 from 197 N (100% nucleotide sequence identity) (Table 1). BAV0511 shows similarity (88% amino acid identity and 93% similarity of the deduced proteins) to two gene loci in *B. pertussis*: BP1629 (GenBank accession number CAE41916) and BP3150 (GenBank accession number CAE43417)^35^. ORFs BP1629 and BP3150 from *B. pertussis* are dehydrogenases involved in the biosynthesis of 2,3-diacetamido-2,3-dideoxy-d-mannuronic acid (D-ManNAc3NAcA)^38^, a component of LPS in *Bordetella*^20,36^. In *B. avium,* the gene locus BAV0511 is represented by only one copy (data not shown). However, Westman et al. 2008 showed that the aforementioned gene loci, BAV0511, BP1629, and BP3150, are involved in forming LPS components in *Bordetella*^38^. Inactivation of BAV0511 should result in the absence of the eleventh sugar residue in the LPS core oligosaccharide^20^. However, the size of LPS from mutant x1760R was similar to that of LPS from mutant × 590/1aR (Fig. 1A). Therefore, interruption of LPS synthesis in the mutant occurred at an earlier stage, leading to the formation of LPS in x1760R, similar to × 590/1aR containing lipid A and seven sugar residues.

Mutations in the phage-resistant *B. avium* mutants CCUG 13726^T^R and 12/574/1/CR were located in the ORF corresponding to BAV2233 from *B. avium* 197 N (Table 1). BAV2233, in turn, is similar to locus BB3394 from *B. bronchiseptica* RB50 (GenBank accession number CAE33886), with 71% aa identity and 80% similarity^35^. Sisti et al. 2017 found that the LPS profile of a *B. bronchiseptica* BB3394 deletion mutant is indistinguishable from that of the wild-type strain^39^. However, N-acetylgalactosamine-branched sugar substitution was absent in the core^39^. In contrast, the mutation in the *B. avium* BAV2233-corresponding ORF found in the present study resulted in the truncation of LPS, indicating a different role for this protein in *B. avium* LPS biosynthesis. Here, LPS from *B. avium* CCUG 13726^T^R migrated lower than LPS from × 590/1aR and x1760R. This finding suggests that the BAV2233-corresponding ORF plays a role in the earlier steps of LPS biosynthesis compared with BAV2235 and BAV0511.

Mutations in the phage-resistant *B. avium* mutant CCUG 13726^T^RC3 was located in the ORF corresponding to BAV0096 (Table 1). BAV0096 is similar to locus BP0091 from *B. pertussis* (GenBank accession number WP_010929614) with 86% identity and 91% similarity. BP0091 belong to the *wlb* locus (WlbC) that involved in the biosynthesis of 2,3-diacetamido-2,3-dideoxy-d-mannuronic acid (D-ManNAc3NAcA) that is a part of band A trisaccharide in *B. pertussis*^40^. Inactivation of BAV0096 resulted in the absence of the eleventh sugar residue in the LPS core oligosaccharide^20^. Thus, LPS in CCUG 13726^T^RC3 was assumed to consist of lipid A and ten sugar residues.

Mutation in the phage-resistant *B. avium* mutant CCUG 13726^T^RC1 was located in the ORF corresponding to BAV0086 (Table 1). BAV0086 belongs to the gene cluster that is located downstream of the genes responsible for the synthesis of the distal trisaccharide and is most probably responsible for the synthesis of the O-antigen of LPS^20^. Our data indicate that the mutant CCUG 13726^T^RC1 possesses LPS with a size similar to that of the fast-migrating molecular species from CCUG 13726^ T^ (Fig. 3A). Thus, LPS from the CCUG 13726^T^RC1 mutant consists of lipid A and a core region.

**Fig. 4:**
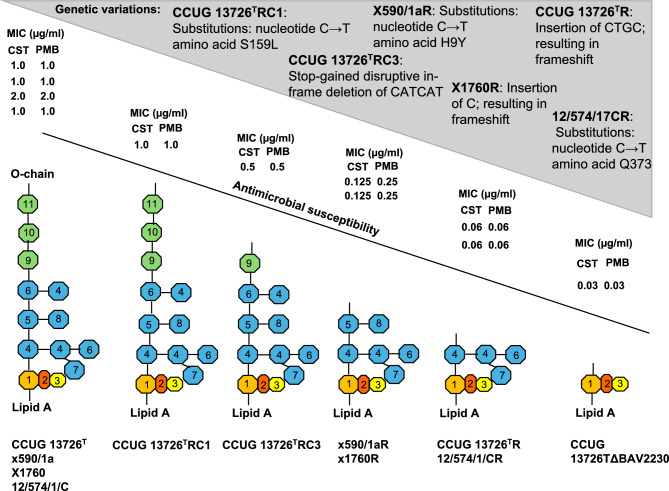
Graphic model showing the LPS molecule of *B. avium* strains and phage-resistant *B. avium* mutants along with the susceptibility to colistin and polymyxin B and the genetic variations. Minimal inhibitory concentration (MIC (µg/ml)), CST- colistin, PMB- polymyxin B; 1) 3-deoxy-D-manno-oct-2-ulosonic acid (KDO); 2) phosphate (P); 3) phosphoethanolamine (PEA); 4) heptose (Hep); 5) glucose (Glc); 6) glucosamine (GlcN); 7) glucuronic acid (GlcA); 8) galactosaminuronic acid (GalNA); 9) β-L-2-acetamido-4-methylamino-fucose (Fuc2NAc4NMe); 10) β−2-acetamido-3-acetamido-2,3-dideoxy-mannuronic acid (Man2NAc3NAcA); 11) α-N-acetyl-GlcN (GlcNAc).

Bacteria modify phage receptors as an important defence mechanism against phage infection^5,15,20^. The genetic variations observed in the *B. avium* mutants investigated in this study resulted modifications in LPS structure, which subsequently led to a decrease in phage adsorption (Fig. 3C and 1D, Fig. 4). However, the CCUG 13726^T^RC1 mutant, containing LPS with a full-size core region, was still resistant to phage infection. Therefore, the receptor for *B. avium* phages is located in the O-antigen region of the LPS.

Bacterial resistance to phages often entails fitness costs, defects in host colonization, and decreased virulence^41–43^. Nevertheless, it has been shown that the lack of motility and inability to detect flagella in vitro at 42 °C (the body temperature of turkeys) is not crucial for *B. avium* infection in birds, although the role of these factors cannot be ignored in the environmental context^44^. Three of the four phage-resistant mutants investigated in more detail showed significantly reduced swimming motility (Table S3 and Fig. S4), while mutant x1760R exhibited a complete loss of motility (Table S3 and Fig. S4). The gene locus equivalent to BAV0511, which was mutated in x1760R, encodes a protein exhibiting 80% aa identity and 89% similarity to the WbpO protein (WP_012613866) found in *Pseudomonas aeruginosa*. In *P. aeruginosa* PAK, this protein is required for the glycosylation of flagellins^45^. The lack of proper flagellin glycosylation leads to defects in the motility of various bacterial species, such as *Burkholderia pseudomallei*, *Burkholderia thailandensis*, and *Helicobacter pylori*^46–48^. Therefore, the involvement of the BAV0511-encoded protein in the glycosylation of flagellin can be assumed, and its inactivation may contribute to the observed defects in the swimming motility of *B. avium*.

Mutations that confer bacterial phage resistance are typically associated with certain constraints. This involves a compromise between the benefits of avoiding phage infection and the expense of overall bacterial fitness^42^. Nevertheless, we did not observe such a loss in fitness in phage-resistant mutants of *B. avium.* Analysis of growth curves performed under optimal growth conditions for *B. avium* showed that phage-resistant mutants had no noticeable changes in their growth characteristics compared to parental isolates (Fig. S2). However, accumulation of phage-resistant mutants after phage infection might lead to changes in the bacterial community^49^. Therefore, co-culture growth competition assays were performed to determine whether the parental *B. avium* isolates were affected by the presence of phage-resistant mutants. Indeed, we did not observe significant differences in the growth characteristics between the parental isolates and their phage-resistant mutants in mixed cultures (Fig. S3); therefore, it does not seem that phage-resistant *B. avium* is outcompeted in a shared habitat, at least under laboratory conditions.

LPS, a central molecule in the outer membrane of Gram-negative bacteria, plays a significant role in maintaining outer membrane integrity^50^. For example, it was previously shown that truncation of LPS increases the susceptibility of mutants to the surfactant SDS and sodium deoxycholate and may alter susceptibility to polymyxin antibiotics^51,52^. In the course of the present study, we could demonstrate that the phage-resistant mutants exhibited an increased susceptibility to colistin and polymyxin B (Table 2). However, due to the lack of *B. avium* breakpoints for these antibiotics, it cannot be determined whether this is a change from resistant to susceptible or merely a change to lower MIC values within the susceptible category. Similarly, it has been previously shown that phage-resistant mutants of *E. coli*^53^, *Acinetobacter baumannii*^54^, and *P. aeruginosa*^55^ are more susceptible to colistin. Although changes in the bacterial cell wall have been assumed in these studies as a causative mechanism, the role of LPS and its changes have remained unclear. However, characterization of the LPS changes and complementation experiments clearly revealed the underlying role. As shown previously, the length of the O-antigen chain of LPS is very important for the protection of bacteria against environmental threats^56^ and for promoting resistance to polymyxin B^57^ and colistin^58^. Here, we demonstrated that the susceptibility of *B. avium* to colistin and polymyxin B is determined by the length of the polysaccharide chain within LPS (Fig. 4). Conversely, LPS can protect bacteria from colistin and polymyxin B, even though the outer membrane is disturbed.

It has previously been figured out that smooth LPS exhibits considerably more proinflammatory activity than rough LPS in vivo, even when administered at equivalent molar concentrations^59^. Hence, phage-resistant *B. avium* mutants are not only more susceptible to antibiotics, but are probably also less virulent. Thus, the study of phage-resistant mutants can help to identify new genes or reveal the function of genes, as well as the nature of the interaction between phages and bacteria^60,61^. Moreover, this may help to clarify the involvement of genes in bacterial virulence^62–64^.

Overall, phage-resistant *B. avium* mutants exhibited increased susceptibility to colistin and polymyxin B, influenced by mutations in genes associated with LPS biosynthesis. Truncation of LPS as a mechanism of phage resistance may also affect virulence. The results of our initial study and the present study, with current findings on the absence of superinfection immunity in phages, the non-transmission of resistance and virulence genes, the absence of increased virulence under laboratory conditions, and the increased sensitivity of phage-resistant mutants to polypeptide antibiotics suggest that the use of phages against *B. avium* is promising^19^. However, since temperate *B. avium* phages do not appear to be suitable for clinical use, the development of genetically modified phages based on these *B. avium* phages could be a promising direction for the future of phage application.

## Material and methods

### Bacterial strains, culture conditions and bacteriophages

*Bordetella avium* isolates used in this study included the type strain CCUG 13726^ T^ (Culture Collection University of Gothenburg) and field isolates × 1760, × 590/1a, and 12/574/1/C^9^. *B. avium* was cultured on tryptic soy agar (TSA; Chemsolute, Renningen, Germany) or tryptic soy broth (TSB; Chemsolute, Renningen, Germany) at 37 °C in ambient air for 18–20 h. *B. avium* bacteriophages vB_BaM-IFTN1 to vB_BaM-IFTN7^19^ were stored at stock concentrations of 2 × 10^9^–2 × 10^10^ pfu/ml at 4 °C in SM buffer (100 mM NaCl, 8 mM MgSO4, 50 mM Tris–HCl pH 7.5, and 0.01% gelatin).

### Isolation of phage-resistant *B. avium* mutants

To isolate phage-resistant mutants, *B. avium* type strain CCUG 13726^ T^ and field isolates × 1760 and × 590/1a were infected with phage vB_BaM-IFTN4, and *B. avium* isolate 12/574/1/C with phage vB_BaM-IFTN3 in 2 ml TSB and incubated at 37 °C for 48 h. To achieve this, bacteria were concentrated to 10^8^ cfu/ml and infected with 20 µL of a phage suspension with a titre of 10^10^ pfu/ml. After incubation, four tenfold serial dilutions were prepared, and 50 μl of each dilution was plated onto dishes with TSA. The plates were then incubated at 37 °C for 24 h in ambient air. Individual colonies were collected and passaged twice on TSA plates (incubated at 37 °C for 24 h). The passaged *B. avium* colonies were evaluated for their susceptibility to phages vB_BaM-IFTN1 to vB_BaM-IFTN7 by a spot assay test on a double agar overlay^19,65^.

### Growth curves of phage-resistant mutants

Growth curves of *the B. avium* parent isolates and their phage-resistant mutants were compiled using optical density (OD_600_) measurements. The bacteria were plated on TSA plates and incubated at 37 °C for 24 h. Then, 2–4 colonies were rubbed into TSB and adjusted to an initial OD_600_ of 0.01. The bacterial suspensions and controls (without inoculated bacteria) were incubated at 37 °C in ambient air in a shaking incubator. The measurement times were 2, 4, 6, 8, 24, 36, 48, 56, and 72 h after inoculation. All data were collected in triplicates.

### Growth competition experiments of *B. avium* isolates and their phage-resistant mutants

To investigate whether phage resistance influences the growth behaviour of *B. avium*, growth competition experiments were performed. Thus, liquid cultures of *B. avium* and their phage-resistant mutants were prepared in TSB and incubated in ambient air at 37 °C in a shaking incubator to an initial OD_600_ of 0.01. Then, the liquid cultures of each *B. avium* isolate were mixed with the cultures of their phage-resistant mutants at a ratio of 1:1 (initial OD_600_ = 0.01), and 20 ml of the mixture was incubated in a shaking incubator at 37 °C. At 0, 4, 8, 24, 48, and 72 h, 50 µl of the bacterial suspension was spread on TSA and TSA agar plates supplemented with colistin at a concentration of 0.06 µg/ml for strains CCUG 13726^ T^/CCUG 13726^T^R and 12/574/1/C/12/574/1/CR, or at a concentration of 0.125 µg/ml for strains × 1769/x1769R and × 590/1a/× 590/1aR. After overnight incubation, the colonies were counted. Differences between the total counts of *B. avium* on TSA agar plates (growth of parent isolates and mutants) and on TSA agar plates supplemented with colistin (only growth of parent isolates) were regarded as the counts of colistin-susceptible *B. avium* mutants. The data were calculated as relative growth curves by performing experiments with two independent replications.

### Construction of trans-complementation plasmids

Native open reading frames (ORFs) were amplified by PCR from *B. avium* CCUG 13726^ T^ chromosomal DNA using S7 Fusion High-Fidelity DNA Polymerase (Biozym Scientific, Germany). For the amplification of the ORFs corresponding to the locus tags in the genome of *B. avium* 197N^22^, the following primers were employed: for the locus tag BAV2233, the primers BAV2233_NI (5’-GCATCATATGAAGACCTCCGCCGGCGCTG-3’) and BAV2233_BI (5’-GCATGGATCCGCAGTTCACGGCTCCGACCC-3’); for BAV0096, the primers BAV0096_NI (5’-GACTCATATGCAATTCATTGATCTGAAAAAA CAGTAC-3’) and BAV0096_BI (5’-GACTGGATCCCTTCATGGGTCAGGCTAAGC-3’); and for BAV0086, the primers BAV0086_NI (5’-GACTCATATGTATCGAAATAACAGA ATCTCTG-3’) and BAV0086_BI (5’-GACTGGATCCGACTAAAGCTTGAATACCCAC-3’). To amplify ORFs corresponding to the loci tags BAV2235 and BAV0511 of *B. avium* 197 N, primers BAV2235_NI (5’-GCATATGAAGATTCTTTACACCAAC-3’) and BAV2235_BI (5’-CGGATCCGATGGGGATGGATGTCATG-3’) and BAV0511_NI (5’-CATATGCGTATTCAAGATGTGAAACTGGCC-3’) and BAV0511_BI (5’-GGATCCGTCGTCATGGTGGCAATTCCTTC-3’), respectively, were chosen. All primers were designed to introduce the recognition sites for the restriction endonucleases NdeI at the 5’-end and BamHI at the 3’-end of the amplified fragment. The amplified fragments were then cloned into the vector pMiniT 2 using the NEB PCR Cloning Kit (New England Biolabs, MA, USA). The recombinant plasmids were digested with the restriction endonucleases NdeI and BamHI, and fragments containing the targeted *B. avium* ORFs were ligated into the corresponding restriction sites of the vector pUR^66^. For complementation analysis, plasmids were introduced into the phage-resistant mutants by tri-parental mating. Conjugative plasmids from *B. avium* isolates × 1760 and × 590/1a were used as helper plasmids.

### Spot assay

The *B. avium* strains were evaluated for susceptibility to phages by spot testing on a double agar overlay. The target bacterial suspension (1.0 mL) (OD600 = 1.0) was mixed with 5 mL of 0.6% TSA soft agar, poured onto a TSA plate, and allowed to solidify. Next, 5 μL of each phage lysate (5 × 10^9^ pfu/ml) was dropped onto the overlay, dried, and cultured at 37 °C for 20 h.

### Inactivation of the ORF corresponding to locus tag BAV2230 in the chromosome of *B. avium* CCUG 13726^ T^ by gene-targeted homologous recombination

The 5’ end (531 bp) and 3’ end (475 bp) of the gene encoding the ORF corresponding to locus tag BAV2230 were amplified by PCR and cloned into the pGEM-T vector, with both ends of the gene being separated by a chloramphenicol resistance cassette. The recombinant plasmid was introduced into *B. avium* strain CCUG 13726^ T^ by electroporation.

### LPS extraction, electrophoresis, and silver staining

LPS was extracted using a Proteinase K microdigestion protocol^67^. Briefly, 2 × 10^8^ CFU of *B. avium* were washed twice with 1 ml of physiological saline (0.9% sodium chloride) and resuspended in 50 µL of lysis buffer (2% w/v SDS, 4% v/v 2-mercaptoethanol, 10% v/v glycerol, 62.5 mM Tris–HCl (pH 6.8), and 0.002% bromophenol blue). Bacteria were incubated at 95 °C for 10 min, cooled to room temperature, and treated with 10 µl of 2.5 mg/ml Proteinase K solution prepared in lysis buffer for 1 h at 56 °C. Five microlitres of each sample were directly loaded onto a 15% SDS polyacrylamide gel (19:1 acrylamide:bisacrylamide). The gels were run at a constant current of 10 mA in Tris–glycine-SDS buffer. The LPS profile was developed according to a previously published protocol^67^.

### Adsorption assay

The ability of phages vB_BaM-IFTN3 and vB_BaM-IFTN4 to adsorb to *B. avium* parent isolates and their phage-resistant mutants was investigated using adsorption assays, as previously described^65^. Briefly, bacteria were resuspended in TSB (adjusted to 5 × 10^8^ cfu/ml) before adding 50 µL of phage lysate (Multiplicity of Infection, MOI = 1.0). The bacteria-phage mix was incubated at 37 °C for 20 min before centrifugation at 17 000 × g for 1 min. The supernatant (100 µL) was diluted in 0.9% sodium chloride solution (NaCl) and used to detect non-adsorbed phages. The resulting plaques of the samples and of a control sample without bacterial cells were counted and evaluated using the double agar overlay plaque assay method^19^. Each assay was independently performed three times.

### Antimicrobial susceptibility testing

Antimicrobial susceptibility testing of the  B. avium type strain CCUG 13726^ T^ and field isolates × 1760, × 590/1a, 12/574/1/C (parent isolates), and their phage-resistant mutants (designated as CCUG 13726^T^R, x1760R, × 590/1aR, 12/574/1/CR) against the antimicrobial agents ampicillin, ceftazidime, ciprofloxacin, colistin, kanamycin, sulfisoxazole, tetracycline, trimethoprim, and polymyxin B was performed. For this purpose, the agar dilution method according to the CLSI document VET01^32^ was used. Briefly, Mueller–Hinton (MH) agar plates (HiMedia Laboratories, Einhausen, Germany) were supplemented with the respective antimicrobial agent in twofold dilution steps starting at 128 µg/ml down to a concentration of 0.002 µg/ml. MH agar plates without antimicrobials were used as growth controls. For inoculum preparation, *B. avium* was cultured overnight on TSA agar plates. Then, four to five colonies were picked and transferred into a 0.9% sodium chloride (NaCl) solution and adjusted to a turbidity equivalent of 0.5 McFarland standard (2 × 10^8^ cfu/ml). The bacterial suspensions were diluted 1:100 in 0.9% NaCl, and a volume of 5 µl of the suspensions was spotted on the agar surface of the MH agar plates to achieve the intended inoculum of 10^4^ cfu/spot and a spot diameter of 5–8 mm. The agar plates were incubated for 16–20 h at 37 °C. As a negative control, the bacterial suspension was spotted onto MH agar plates without antimicrobial agents. As quality control strain, *E. coli* ATCC 25,922 was included in the assays. Endpoints were determined by placing the agar plates on a dark surface and visually observing bacterial growth. The minimum inhibitory concentration (MIC) was defined as the lowest concentration at which bacterial growth was inhibited. All experiments were performed three times independently.

### Phage-resistant *B. avium* mutants selected on colistin- and polymyxin B-supplemented agar plates

To investigate whether it is possible to isolate phage-resistant *B. avium* mutants that did not show increased susceptibility to colistin and polymyxin B, a spot assay of phage vB_BaM-IFTN4 on the type strain CCUG 13726^ T^ was performed. After 24 h of infection, the lysis zone was excised and placed in 2 ml of TSB to elute viable bacteria. Serial dilutions of bacterial suspensions were prepared and plated on TSA agar plates with colistin (0.2 µg/ml) or polymyxin B (0.2 µg/ml). After 48 h of incubation at 37 °C, single colonies were selected and tested for resistance to phages vB_BaM-IFTN1 to vB_BaM-IFTN7. Mutants selected on colistin-supplemented agar plates were designated as CCUG 13726^T^RC (1 to 8), and the mutants selected on polymyxin B agar plates as CCUG 13726^T^RP (1 to 8).

### Swimming agar assay

A swimming agar assay to determine the motility of *B. avium* was performed using a plate-based method, as previously described for *Pseudomonas aeruginosa*^68^. Sterile toothpicks were used to puncture the 0.3% TSA layer. The plates were incubated in an upright position at 37 °C for 24 h.

### Whole-genome sequence analysis and nucleotide sequence deposit

Short-read, paired end sequencing of *B. avium* genomes was performed at the Federal Institute for Risk Assessment (BfR), Berlin, Germany. The genomic DNA libraries for sequencing were prepared using the Nextera XT Library Prep kit (Illumina, CA, USA) according to the manufacturer’s recommendation. Libraries were purified using the Mag-Bind RXNPure Plus magnetic beads (Omega Biotek), following the instructions provided by the manufacturer. Then, libraries were pooled in equimolar amounts according to the quantification data provided by the Qubit dsDNA HS Assay (Thermo Fisher Scientific). Thereafter, the libraries were sequenced in an Illumina NextSeq 500 platform, obtaining 100 to 150 bp paired-end reads which were trimmed (Trim Galore 0.6.0) and filtered according to quality criteria (FastQC 0.11.9). Single nucleotide polymorphisms between the phage-resistant mutants and parent isolates were identified using Snippy v4.6.0^25^. The nucleotide sequences of the phage-resistant mutants were deposited in the GenBank database under the accession numbers JAWHTY000000000 (12/574/1/CR), JAWHTZ000000000 (× 1760), JAWHUA000000000 (x1760R), JAWHUB000000000 (CCUG 13726^T^R), JAWHUC000000000 (× 590/1a), and JAWHUD000000000 (× 590/1aR), JBBEUU000000000 (CCUG 13726^T^RC1), JBBEUV000000000 (CCUG 13726^T^RC3).

### Statistical analysis

Microsoft Excel 2016 was used to calculate mean values, standard deviations from experimental results, and statistical significances of differences in results (Student´s *t-*test). Statistical significance was set at *P* < 0.05.

## Supplementary Information

Supplementary Information.

## Data Availability

Data is provided within the manuscript or supplementary information files. In addition, the nucleotide sequences were deposited in the GenBank database under the accession numbers JAWHTY000000000, JAWHTZ000000000, JAWHUA000000000, JAWHUB000000000, JAWHUC000000000, JAWHUD000000000, JBBEUU000000000, and JBBEUV000000000.
