# Supplementary material for: Phage resistance of Bordetella avium due to an altered cell wall results in increased susceptibility to the polypeptide antibiotics colistin and polymyxin B
Source: Sci Rep. 2025 Dec 19;15:44227. doi: 10.1038/s41598-025-30405-7 (PMC12722373; doi:10.1038/s41598-025-30405-7)

Table S1. MICs of *B. avium* isolates and their phage-resistant mutants (R) against eight antimicrobial agents.

Isolate	MIC (µg/ml)[a]						
	AMP	CTZ	CIP	K	SF	TMP	TET
**CCUG 13726^{T}**	0.25	0.5	0.5	8	30	2	0.12
**CCUG 13726^{T}R**	0.25	0.5	0.5	8	30	1	0.12
**12/574/1/C**	0.5	1	0.5	8	>128	2	64
**12/574/1/CR**	1	2	0.5	8	>128	2	64
**x590/1a**	0.12	0.25	0.5	8	>128	1	64
**X590/1aR**	0.06	0.25	0.5	4	>128	1	64
**x1760**	0.12	0.5	1	8	>128	>128	128
**x1760R**	0.25	1	1	8	>128	>128	128

[a]MIC (µg/ml) - minimal inhibitory concentration

Antimicrobial agents: AMP-ampicillin, CTZ-ceftazidime, CIP-ciprofloxacin, K-kanamycin, SF-sulfafurazole, TET-tetracycline, TMP-trimethoprim

Table S2. MIC values of *B. avium* isolates and their phage-resistant mutants against novobiocin and rifampicin.

**Isolate**[b]	**MIC (µg/ml)**[a]	
	**NV**	**RD**
CCUG 13726^{T}	**4.0**	**8.0**
CCUG 13726^{T}R	**4.0**	**16.0**
CCUG 13726^{T}RC3	**8.0**	**32.0**
CCUG 13726^{T}RC1	**8.0**	**32.0**
CCUG 13726^{T}Δ2230	**0.25**	**0.5**
12/574/1/C	**1.0**	**4.0**
12/574/1/CR	**2.0**	**8.0**
x590/1a	**2.0**	**8.0**
X590/1aR	**4.0**	**16.0**
x1760	**1.0**	**4.0**
x1760R	**1.0**	**8.0**

[a]minimal inhibitory concentration
NV- novobiocin, RD- rifampicin
[b] RC mutants were selected at low frequency on colistin supplemented agar plates.

Figure S1. Host range determination. The collection of *B. avium* type strain CCUG 13726^{T}, *B. avium* isolates 12/574/1/C, X1760 and X590/1a and of phage-resistant mutants (R) was evaluated for susceptibility to phages vB_BaM-IFTN1 to vB_BaM-IFTN7. Black bars indicate lysis and white bars indicate no lysis.

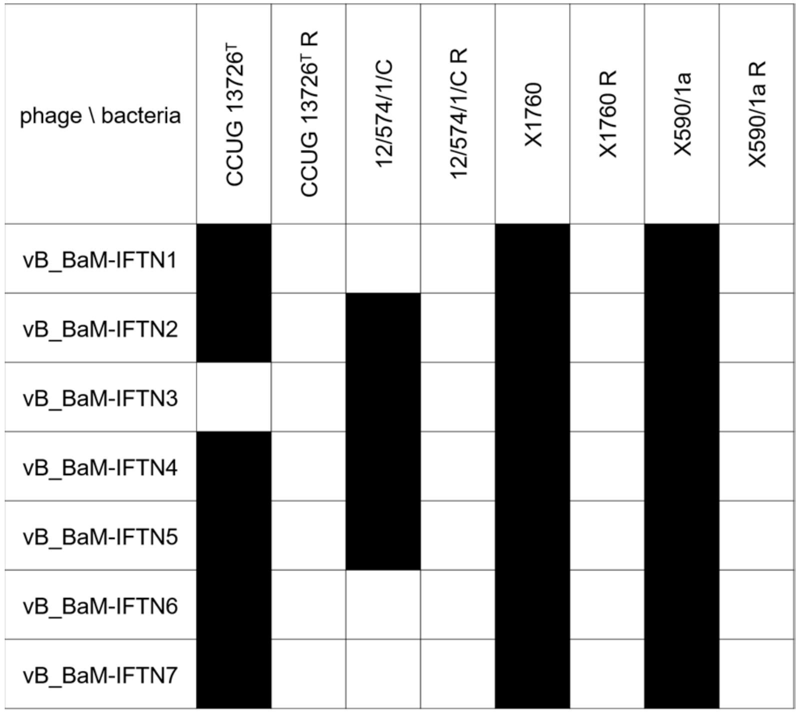

Figure S2. Growth curves with optical density (OD) measurements of *B. avium* isolate 12/574/1/C compared with the phage-resistant mutant 12/574/1/C R (A), and of *B. avium* type strain CCUG 13726^{T} (B), *B. avium* isolates X1760 (C), and X590/1a (D), compared with their phage-resistant mutants CCUG 13726^{T}R (B), X1760R (C) and X590/1aR (D). These data are the average of three independent experiments (mean ± SD).

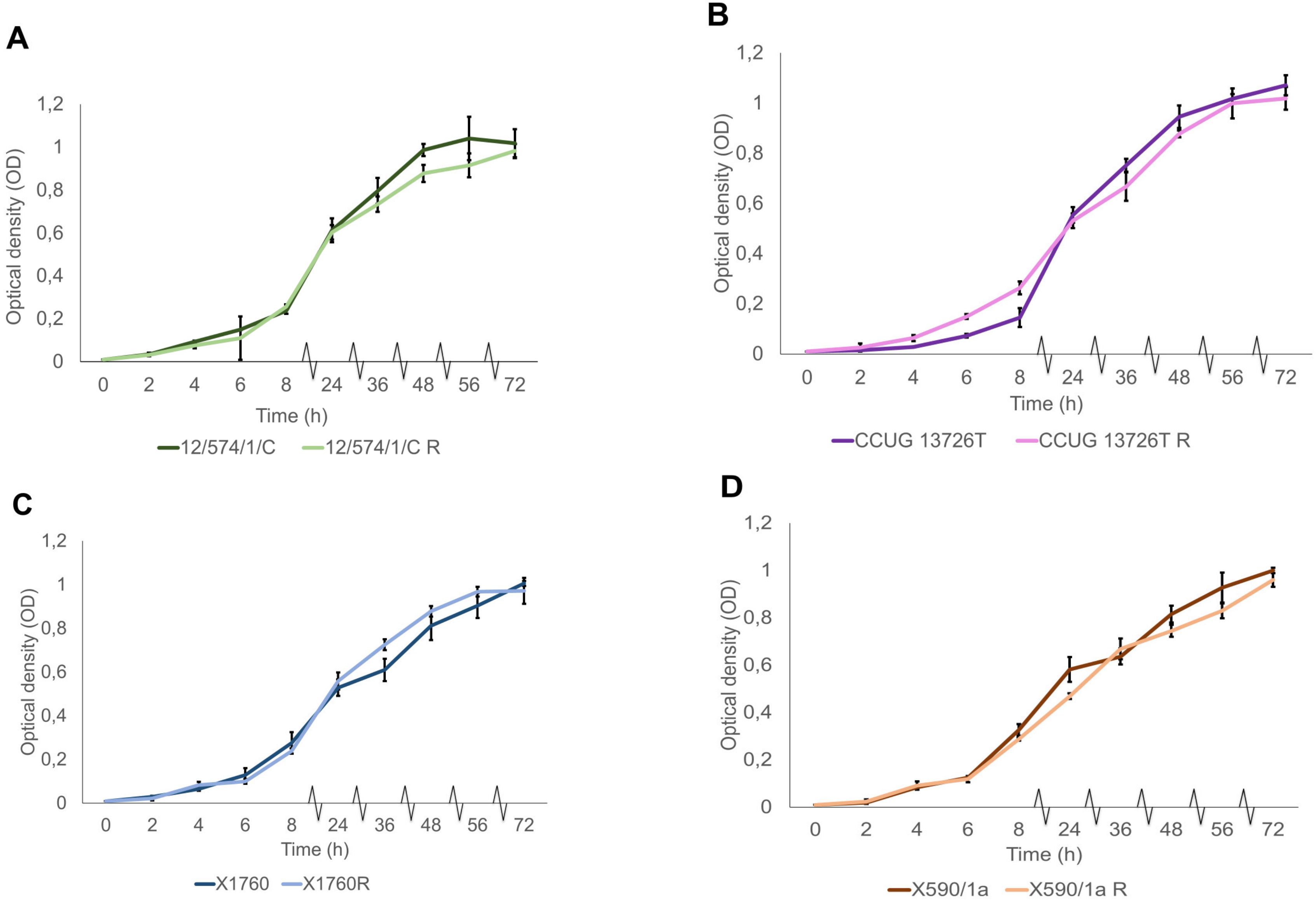

Figure S3. Relative growth curves of *B. avium* isolate 12/574/1/C and mutant 12/574/1/CR (A), *B. avium* type strain CCUG 13726^T and mutant CCUG 13726^{T}R (B), isolate x1760 and mutant x1760R (C) and isolate x590/1a and mutant x590/1aR (D). The results represent the average of one trial in duplicate (mean ± SD).

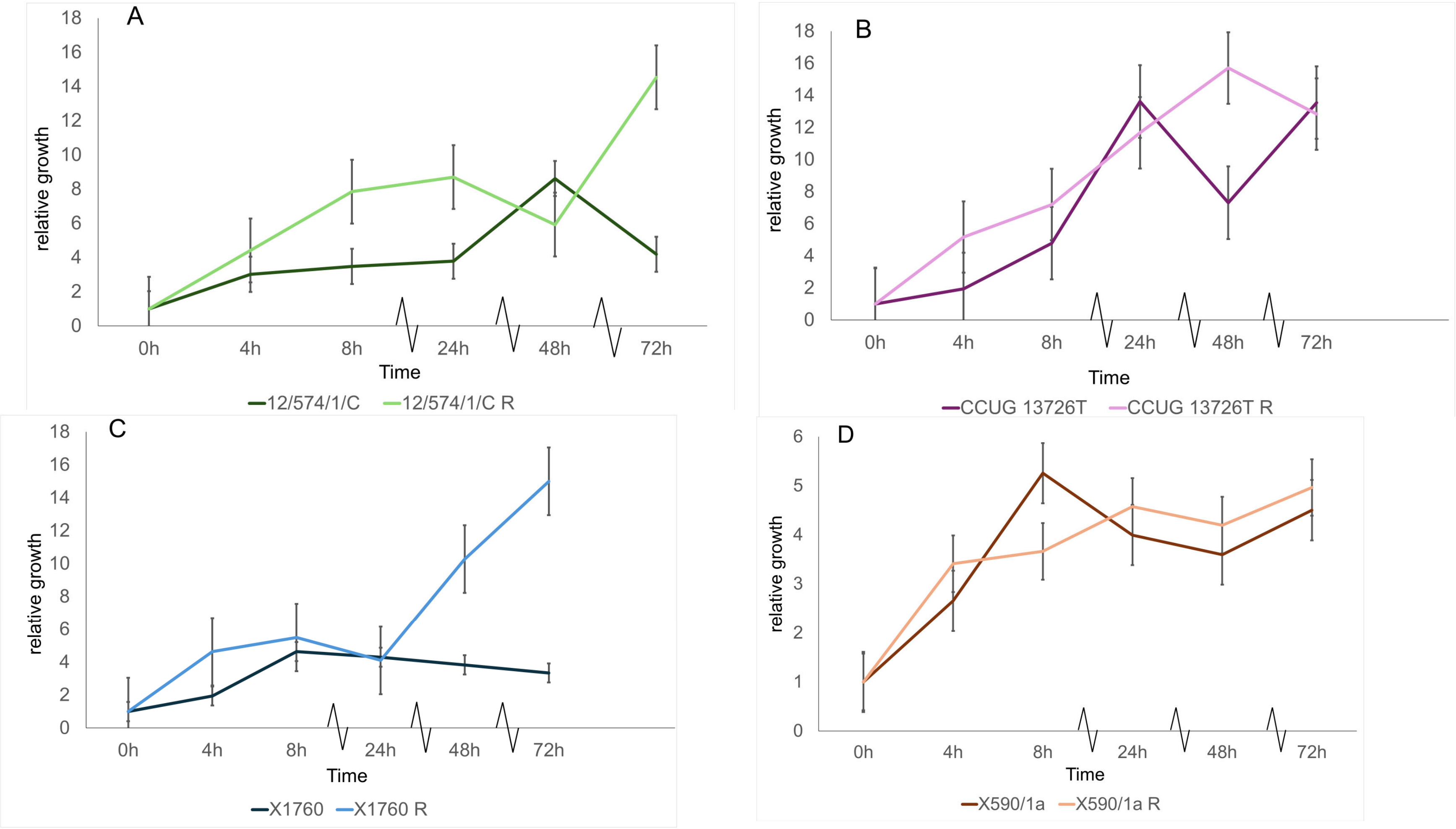

Fig. S4 Swimming motility of *B. avium* phage-resistant mutants and their original strains

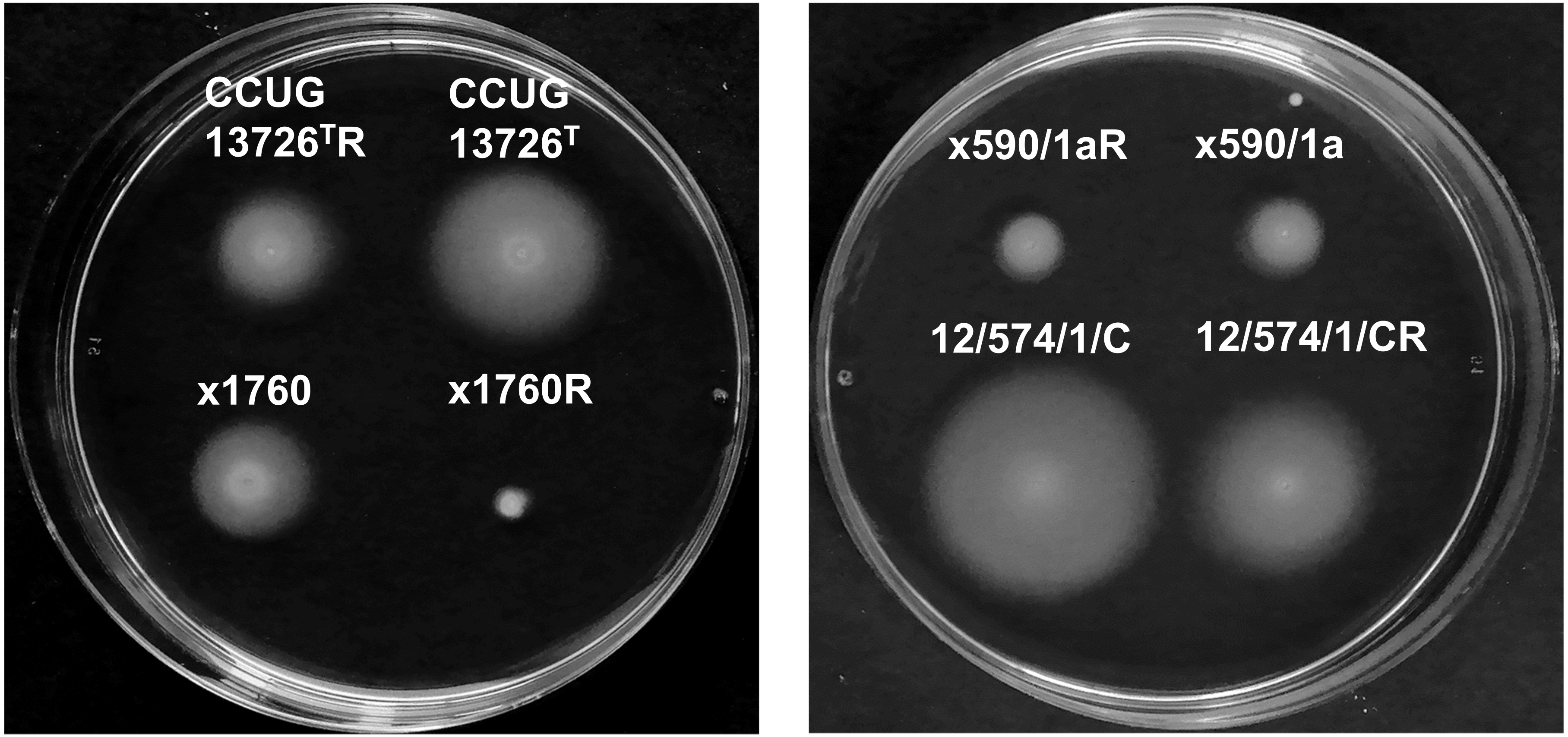

Figure 1A (original image)

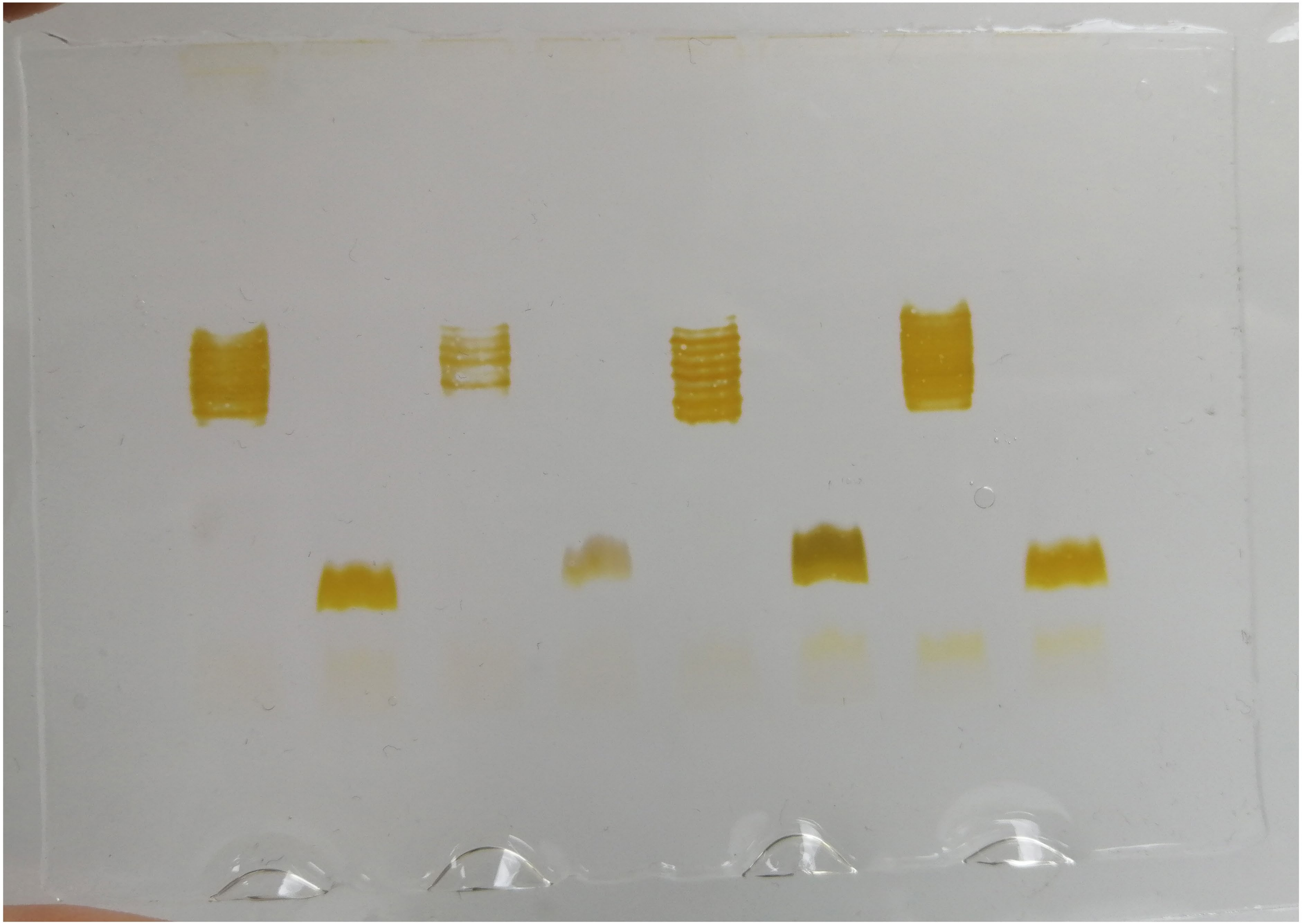

Figure 1B (original image)

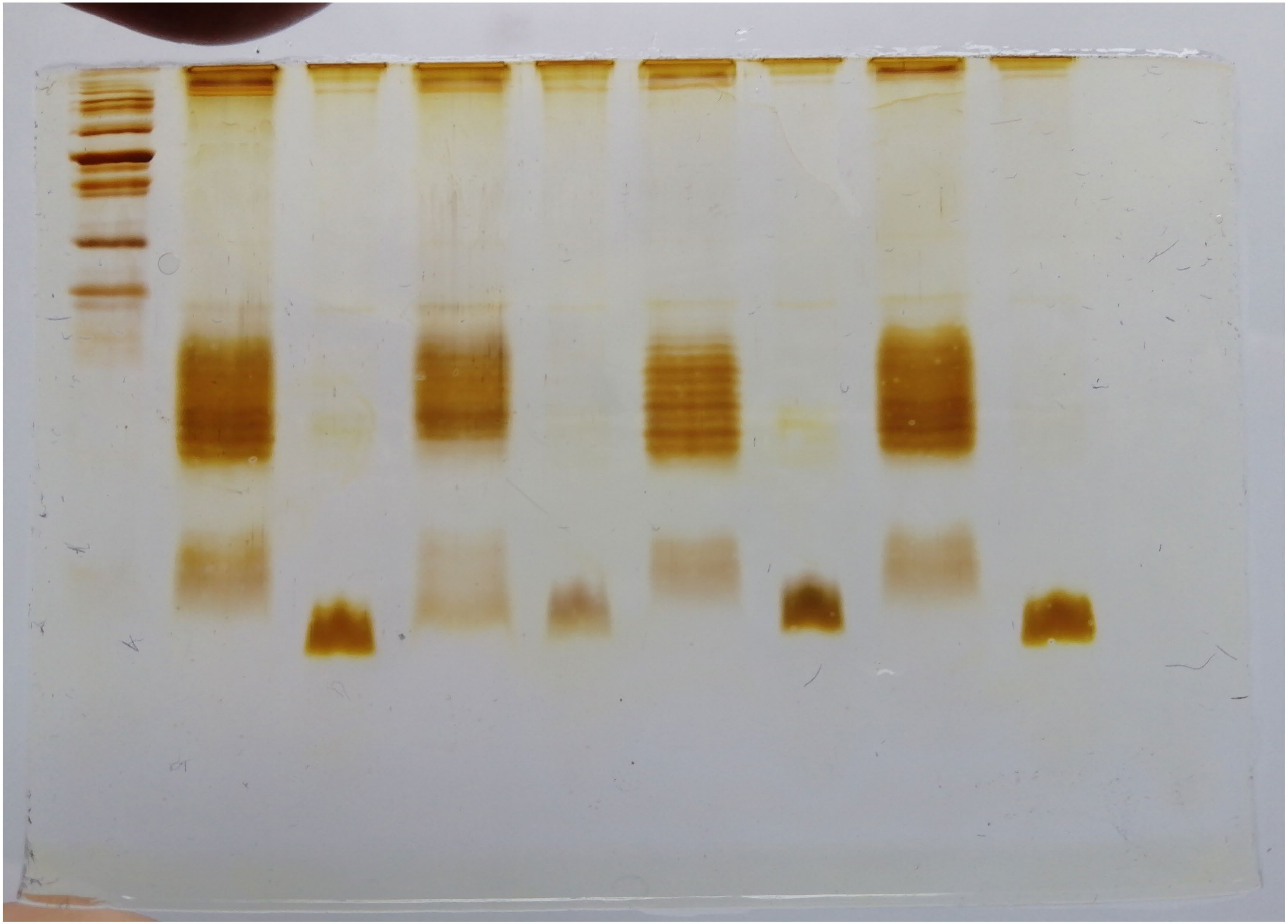

Figure 1C (original image)

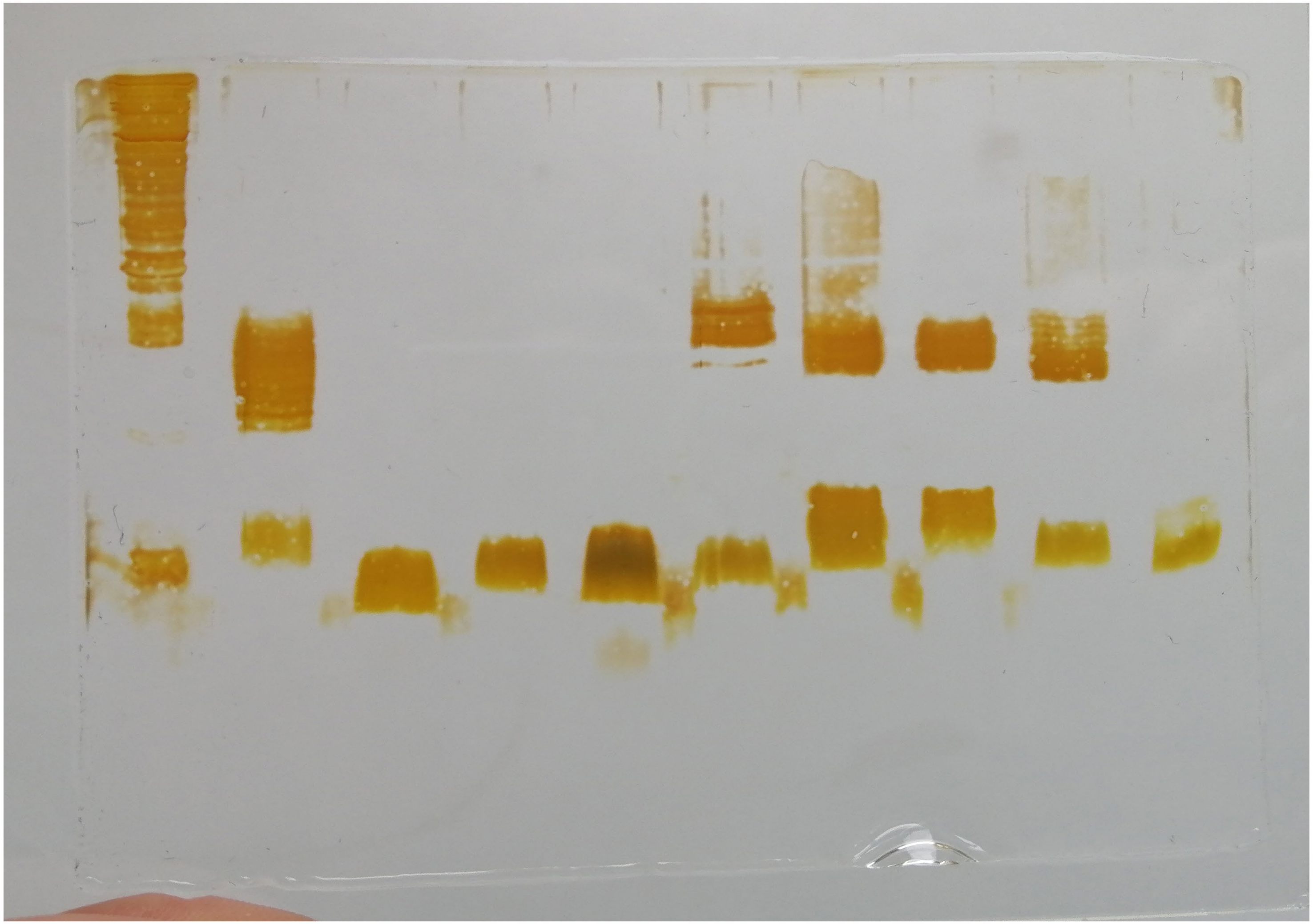

Figure 2B, C, D (original image)

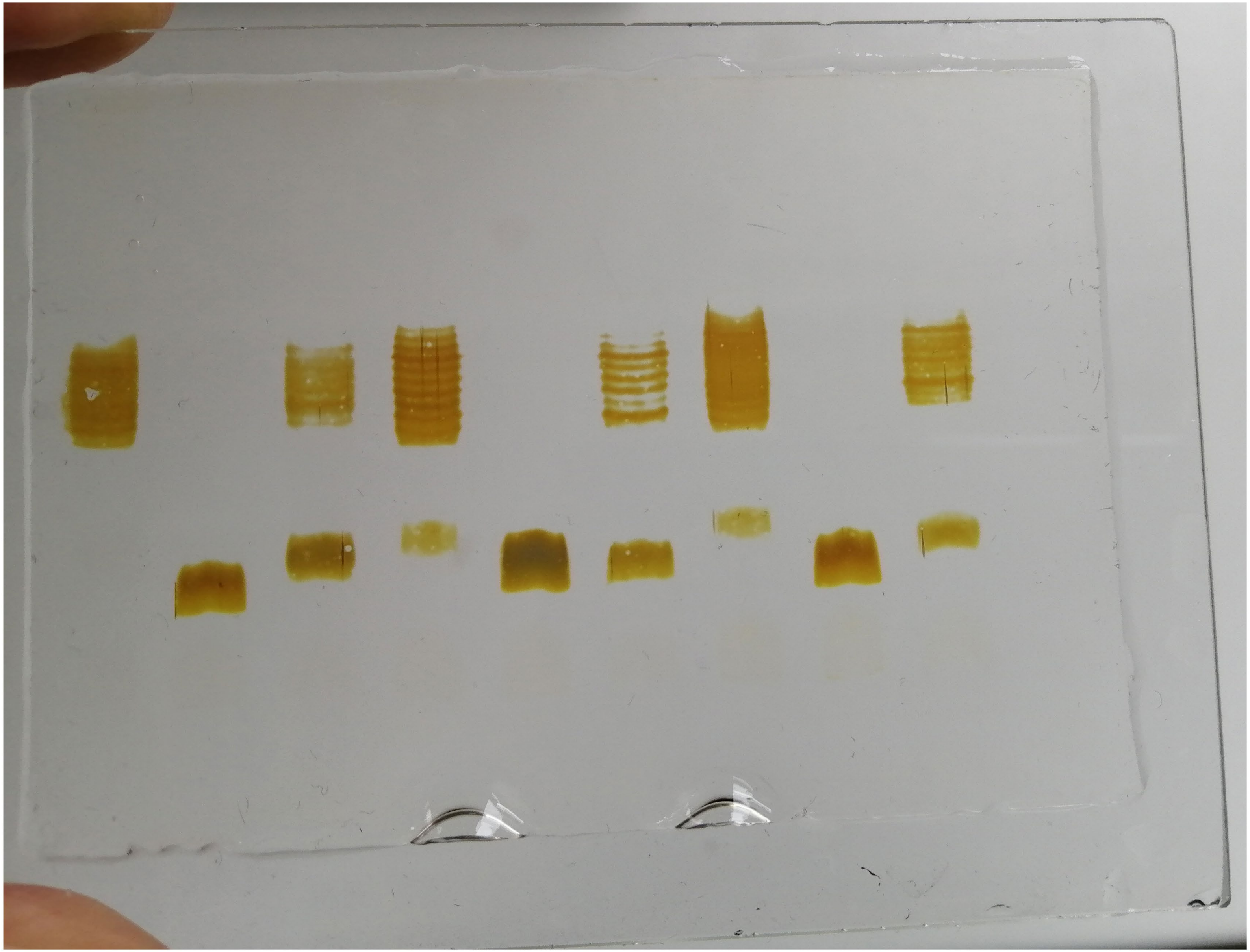

Figure 2E (original image)

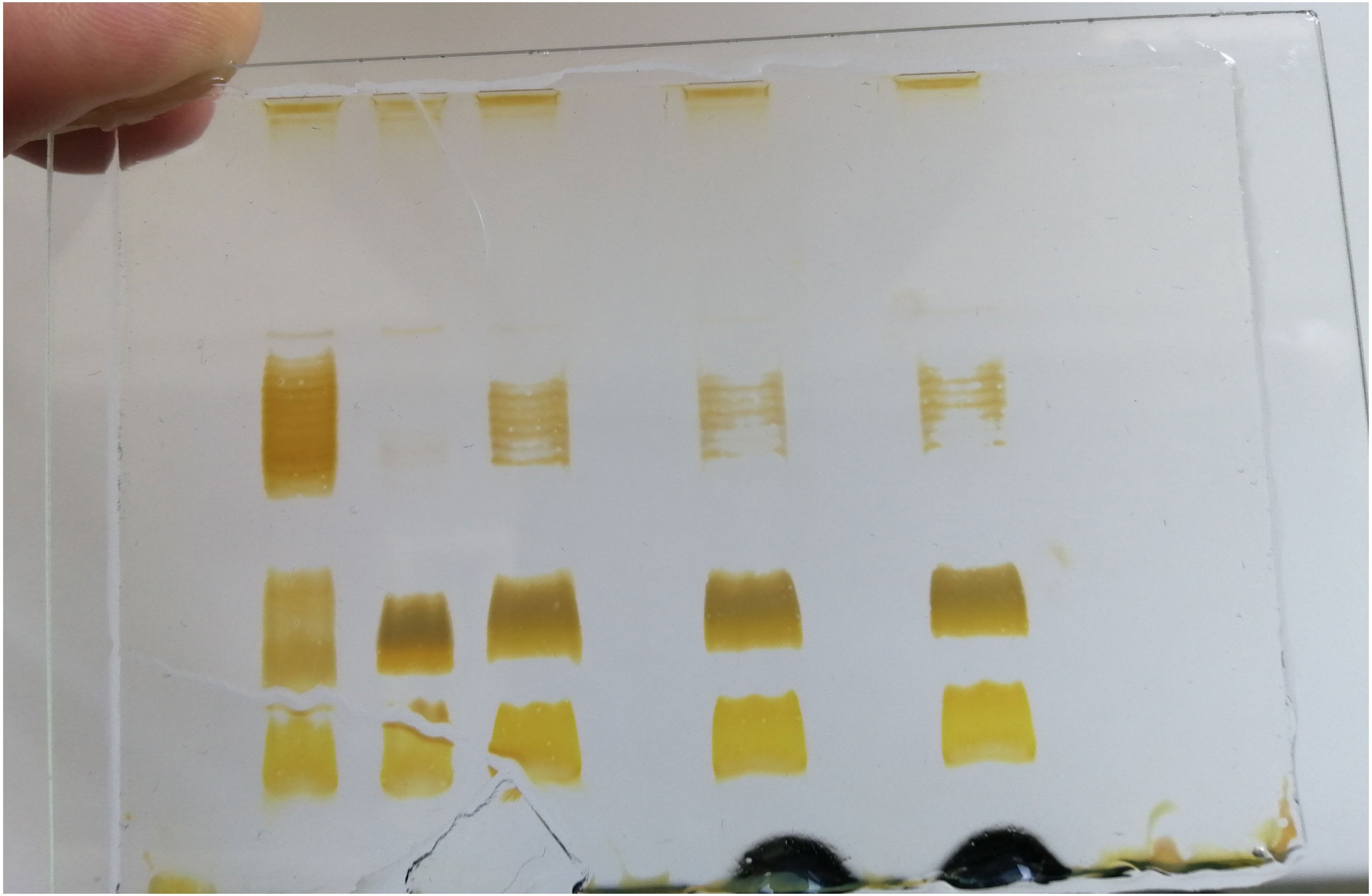

Figure 2F (original image)

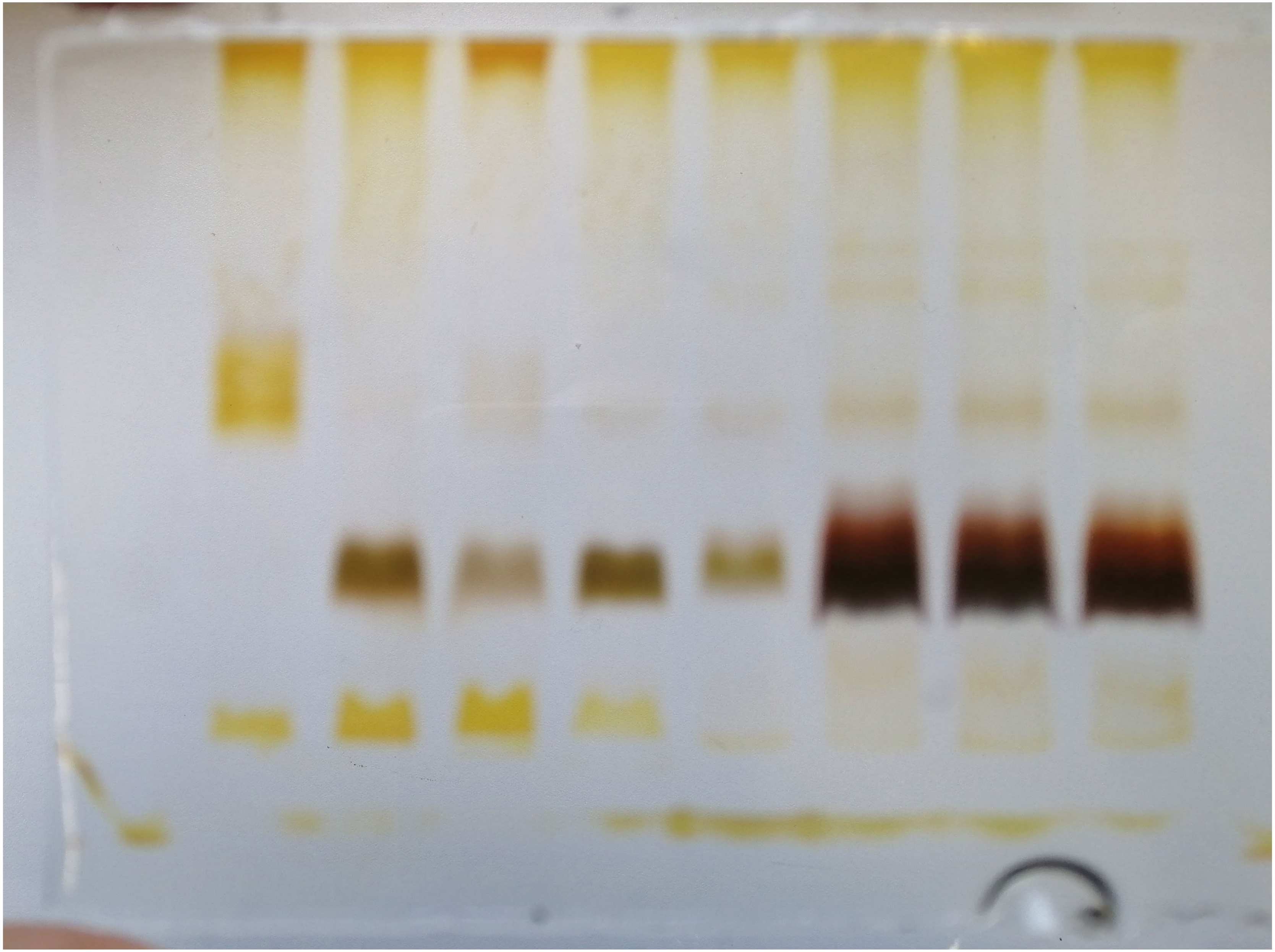

Figure 2G (original image)

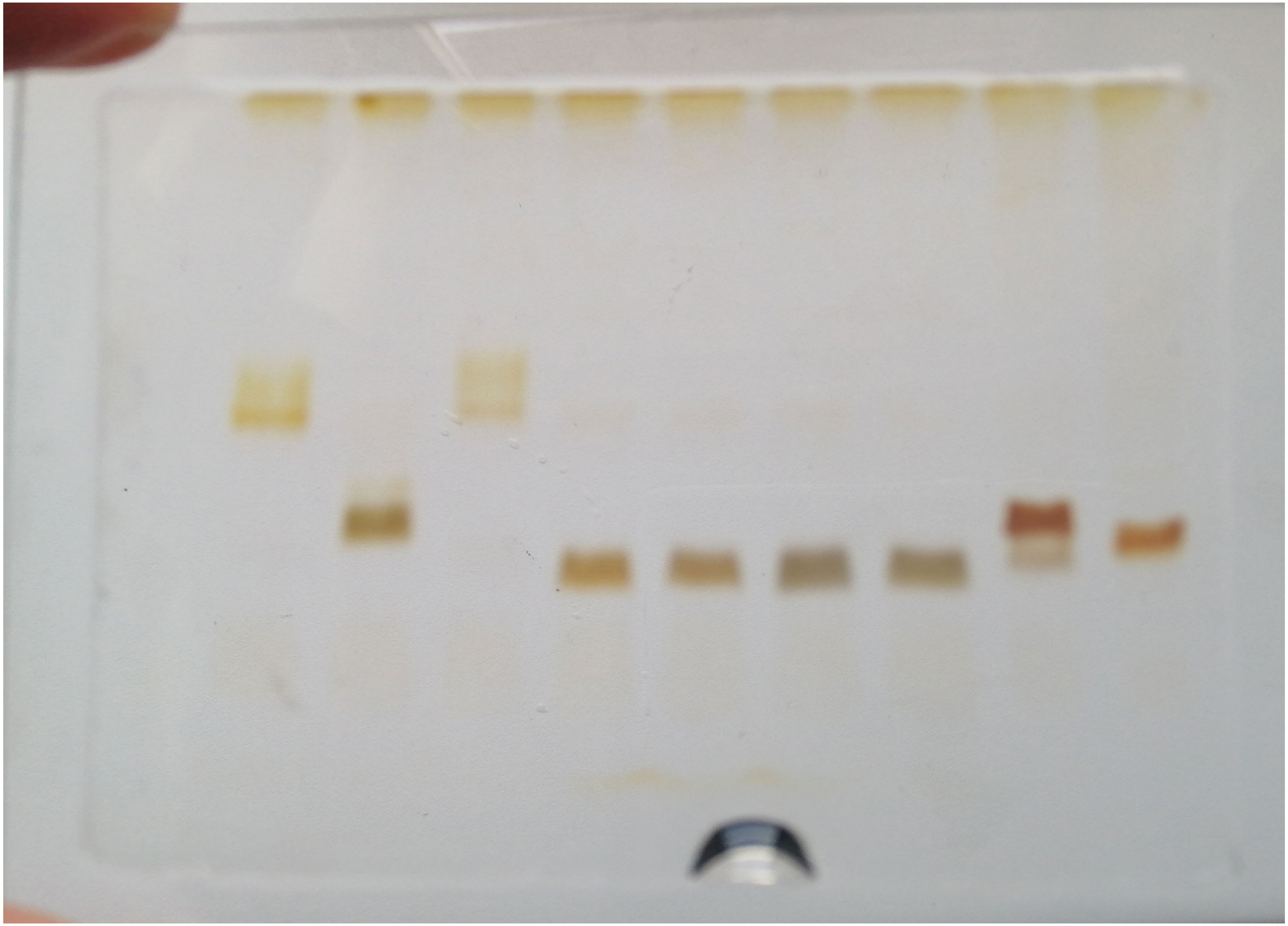

Figure 3A (original image)

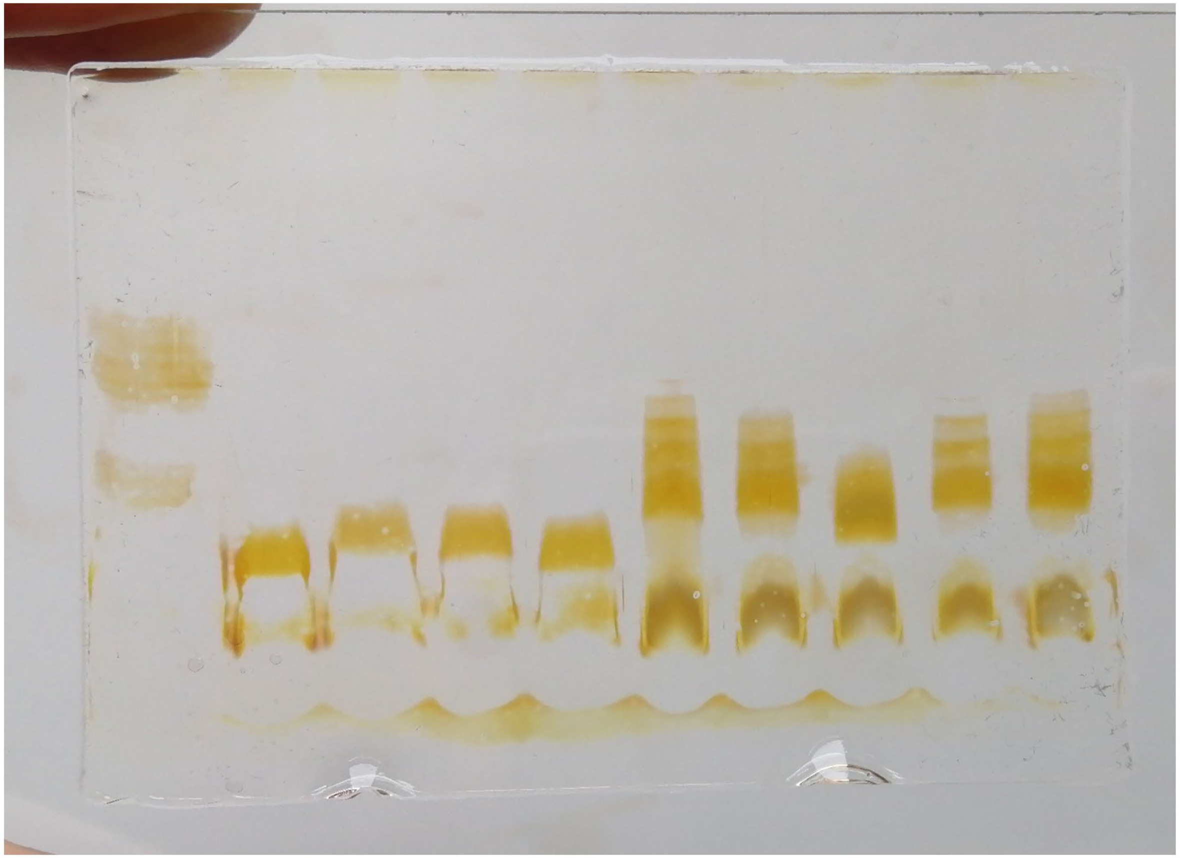

Figure 3B (original image)

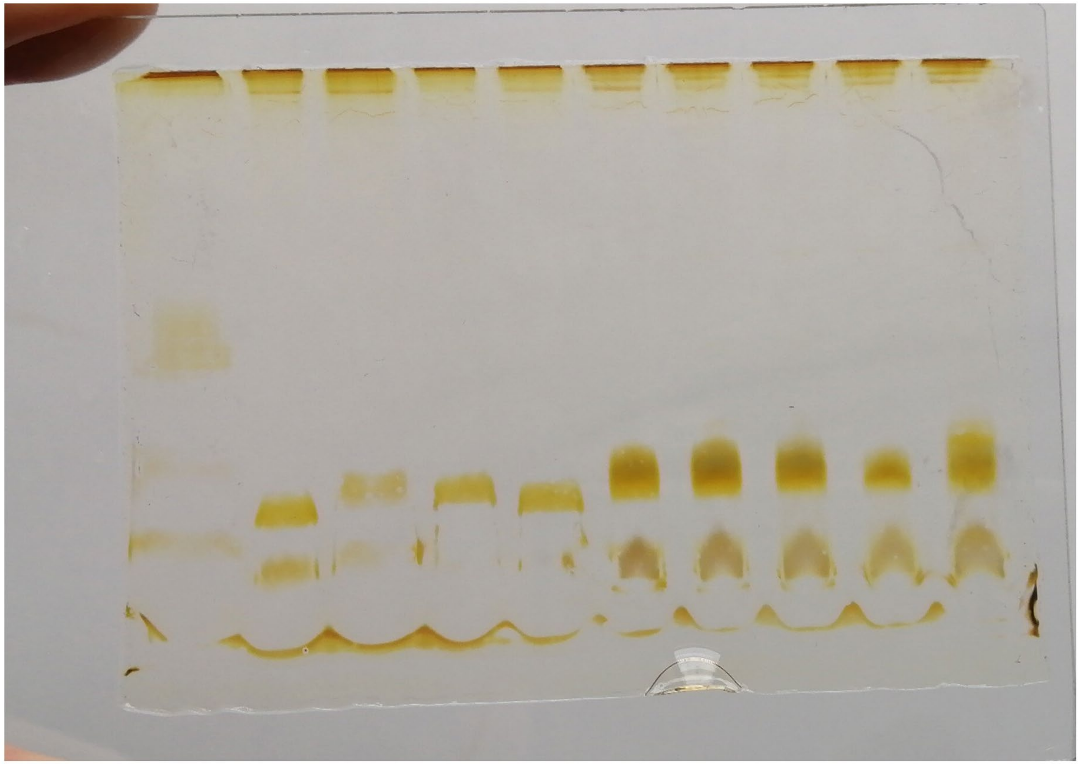

Figure 3C (original image)

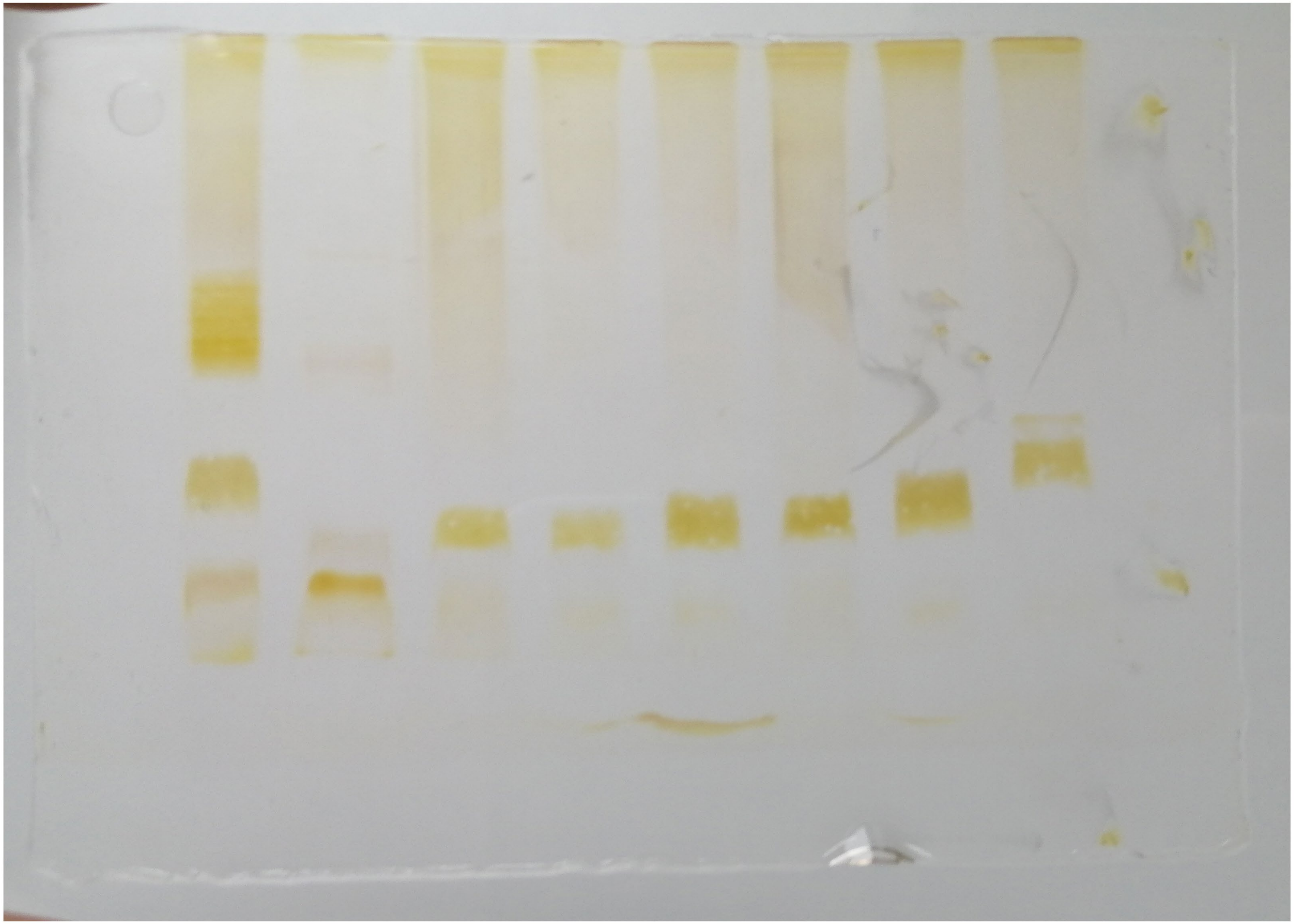

Supplement: Supplementary file 1 — Supplementary Information. [file 41598_2025_30405_MOESM1_ESM.pdf]
